# Two herpesviral noncoding PAN RNAs are functionally homologous but do not associate with common chromatin loci

**DOI:** 10.1371/journal.ppat.1007389

**Published:** 2018-11-01

**Authors:** Johanna B. Withers, Eric S. Li, Tenaya K. Vallery, Therese A. Yario, Joan A. Steitz

**Affiliations:** 1 Department of Molecular Biophysics and Biochemistry, Boyer Center for Molecular Medicine, Yale University School of Medicine, New Haven, Connecticut, United States of America; 2 Howard Hughes Medical Institute, Boyer Center for Molecular Medicine, Yale University School of Medicine, New Haven, Connecticut, United States of America; University of California, Berkeley, UNITED STATES

## Abstract

During lytic replication of Kaposi’s sarcoma-associated herpesvirus (KSHV), a nuclear viral long noncoding RNA known as PAN RNA becomes the most abundant polyadenylated transcript in the cell. Knockout or knockdown of KSHV PAN RNA results in loss of late lytic viral gene expression and, consequently, reduction of progeny virion release from the cell. Here, we demonstrate that knockdown of PAN RNA from the related Rhesus macaque rhadinovirus (RRV) phenocopies that of KSHV PAN RNA. These two PAN RNA homologs, although lacking significant nucleotide sequence conservation, can functionally substitute for each other to rescue phenotypes associated with the absence of PAN RNA expression. Because PAN RNA is exclusively nuclear, previous studies suggested that it directly interacts with host and viral chromatin to modulate gene expression. We studied KSHV and RRV PAN RNA homologs using capture hybridization analysis of RNA targets (CHART) and observed their association with host chromatin, but the loci differ between PAN RNA homologs. Accordingly, we find that KSHV PAN RNA is undetectable in chromatin following cell fractionation. Thus, modulation of gene expression at specific chromatin loci appears not to be the primary, nor the pertinent function of this viral long noncoding RNA. PAN RNA represents a cautionary tale for the investigation of RNA association with chromatin whereby cross-linking of DNA spatially adjacent to an abundant nuclear RNA gives the appearance of specific interactions. Similarly, PAN RNA expression does not affect viral transcription factor complex expression or activity, which is required for generation of the late lytic viral mRNAs. Rather, we provide evidence for an alternative model of PAN RNA function whereby knockdown of KSHV or RRV PAN RNA results in compromised nuclear mRNA export thereby reducing the cytoplasmic levels of viral mRNAs available for production of late lytic viral proteins.

## Author summary

Herpesviruses produce noncoding RNAs, some of which are essential to the viral life cycle. One such noncoding RNA from Kaposi’s sarcoma-associated herpesvirus is the polyadenylated, nuclear (PAN) RNA, which is required for production and release of progeny virions from infected cells. In this study, we demonstrate that although lacking nucleotide sequence conservation, PAN RNAs from two related viruses–when knocked down–exhibit the same phenotype, loss of late lytic viral gene expression and progeny virion production. Moreover, they can functionally substitute for each other to rescue this phenotype. We demonstrate that, in contrast to published literature, the reduction in viral gene expression upon PAN RNA knockdown is not due to loss of PAN RNA association with conserved, specific chromatin loci, nor does PAN RNA expression affect the viral transcription factor complex required for generation of the late lytic viral mRNAs. We present data suggesting that PAN RNA instead serves as a binding platform to sequester cellular proteins that are mislocalized to the nucleoplasm. These herpesviral noncoding RNAs can serve as models for the mechanistic study of human noncoding RNAs.

## Introduction

Kaposi’s sarcoma-associated herpesvirus (KSHV) is an opportunistic pathogen of human immunodeficiency virus (HIV) patients and the etiological agent of several human cancers, including Kaposi sarcoma and primary effusion lymphoma [1]. The KSHV life cycle includes a latent phase, when viral gene expression is largely absent and no progeny virions are produced, and a lytic phase, characterized by robust viral gene expression and virus replication. The most abundant lytic phase viral RNA is a 1 kb, noncoding, polyadenylated nuclear RNA called PAN [2–4]; PAN RNA accounts for up to 80% of the polyadenylated RNA present in a lytically infected cell. A 3′-end element called the element for nuclear expression (ENE) is required to maintain PAN RNA at these elevated levels. Crystallographic analysis of the PAN ENE complexed with an A9 oligonucleotide revealed that the U-rich internal loop of the ENE forms a triple-stranded interaction with the poly(A) tail of its own transcript [5]. This triple-helical RNA structure that shields the 3′ end [6–8] robustly inhibits nuclear RNA decay of PAN RNA.

Although the ENE has been well studied, we still know little about the function of PAN RNA. Previous work has demonstrated that loss of PAN RNA, either through genetic knockout from the viral genome or antisense depletion of the transcript, results in misregulation of late lytic viral genes and host immune response genes [2, 9]. The accompanying reduction in virion release upon KSHV PAN RNA knockdown highlights its essential role during the lytic phase, but the mechanism underlying the phenotypes associated with loss of PAN RNA is unknown.

PAN RNA is exclusively nuclear, which prompted efforts to demonstrate that KSHV PAN RNA associates directly with the human and viral genomes [10, 11]. A potential mechanism for PAN RNA function emerged from two studies: interaction of KSHV PAN RNA with viral latency-associated nuclear antigen (LANA) [12] and chromatin isolation by RNA purification (ChIRP [13]) studies of KSHV-infected human B-cells [10]. *In vivo* data suggested that PAN RNA may regulate chromatin states by competitively preventing LANA from associating with histone H3, an interaction required for regulating and maintaining latency [12]. On the promoter of the KSHV master lytic activator, RTA/ORF50, PAN RNA was shown to interact with demethylases JMJD3 and UTX [10]. KSHV PAN RNA ChIRP studies extended these conclusions to suggest ubiquitous PAN RNA binding to both the host and KSHV genomes [11]. Thus, the literature posits that PAN RNA regulates late gene expression by a mechanism dependent on chromatin association.

Genes encoding PAN RNA homologs map to syntenic regions within gammaherpesvirus genomes. As is common for many long noncoding RNAs (lncRNAs) [14, 15], PAN RNAs are poorly conserved at the sequence level, but most contain ENEs homologous to that originally identified at the 3′ end of KSHV PAN RNA [16]. Bioinformatic studies revealed ENEs, thus indicating the presence of PAN RNAs, in four other γ-herpesviruses: retroperitoneal fibromatosis-associated herpesvirus Macaca nemestrina (RFHVMn), Rhesus macaque rhadinovirus (RRV), and equine herpesvirus 2 and 5 (EHV-2 and EHV-5) [8]. A fifth PAN RNA homolog lacking an ENE was identified as a highly abundant noncoding RNA expressed from a syntenic genomic locus of bovine herpesvirus 4 (BHV-4) [8, 17].

RRV PAN RNA is a ~1.3-kb transcript found in the nuclei of lytic RRV-infected cells. As is the case for KSHV PAN RNA, the master herpesviral lytic activator ORF50 binds the promoter and activates expression of RRV PAN RNA [8]. Although RRV PAN RNA appears not to contain the small 5′-hairpin motif that binds viral ORF57 in KSHV PAN RNA, the RRV PAN RNA homolog is highly abundant in RRV-infected cells [8]. Moreover, similar to KSHV, RRV PAN RNA binds nuclear relocalized cytoplasmic polyA binding protein (PABPC) and is upregulated by the viral SOX protein in lytically infected cells [2, 8].

KSHV and RRV have well-established cell culture models. The BCBL-1 cell line is a clinical isolate from a body cavity-based lymphoma and is a naturally KSHV-infected human B-lymphocyte cell line. A comparable RRV-infected rhesus B-cell line does not exist; all known rhesus B-lymphocyte lines were immortalized using another human herpesvirus, Epstein-Barr virus (EBV). EBV and KSHV co-infections are known to collude and produce results different from those with KSHV alone [18] and similar interactions would be expected to occur between EBV and RRV. Therefore, RRV is studied instead in the human EBV-negative B-cell line, BJAB, which was de novo infected with RRV [19].

In this study, we demonstrate that knockdown of PAN RNA from the related herpesvirus RRV phenocopies that of KSHV PAN RNA knockdown. Furthermore, despite lacking nucleotide sequence conservation, expression of either KSHV or RRV PAN RNA can rescue production of progeny virions by either KSHV or RRV bacmids lacking the PAN RNA locus. Using CHART (Capture Hybridization Analysis of RNA Targets) [20], we revisited the hypothesis that PAN RNA associates with specific chromatin loci to modulate gene expression during the KSHV lytic phase. Our analysis of PAN RNA from two related viruses, KSHV and RRV, suggests that the primary function of herpesviral PAN RNA is not regulation of gene expression by interacting with specific sites on chromatin. Instead, we find that PAN RNA expression is required for efficient mRNA export from the nucleus. These data emphasize the need for unbiased approaches for ascribing functions to ncRNAs.

## Results

### Knockdown of RRV PAN RNA reduces progeny virion release without altering intracellular viral DNA levels

To determine whether KSHV and RRV PAN RNA homologs perform the same function during the viral life cycle, we characterized the phenotype associated with knocking down RRV PAN RNA expression in lytic BJAB RRV cells. Anti-RRV antibodies useful for investigating the effect of PAN RNA expression on late lytic gene expression are not available at this time; however, quantitative PCR (qPCR) can assess viral DNA replication and virion production to confirm the downstream phenotypic changes associated with KSHV PAN RNA loss [2].

**Fig 1:**
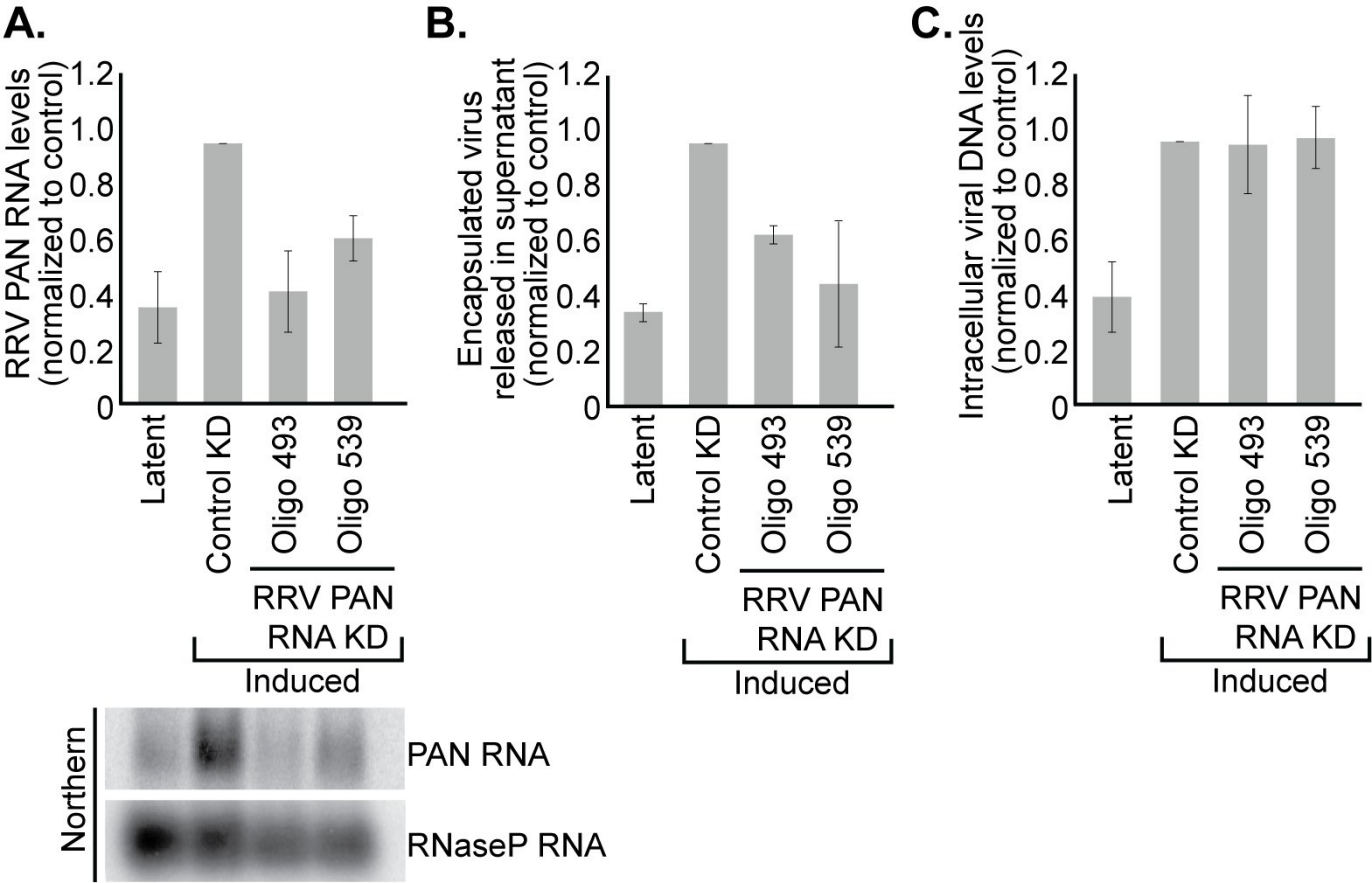
Knockdown of RRV PAN RNA reduces progeny virion release without altering intracellular viral DNA levels. BJAB RRV cells were induced with 100 nM trichostatin-A (TSA) following electroporation with antisense oligonucleotides complementary to either GFP mRNA (control KD) or RRV PAN RNA (Oligo 493 and Oligo 539). (A) Total RNA was processed 40 h after lytic induction. PAN RNA levels were quantified by Northern blot. (B) Seven days after induction, encapsulated viral DNA released into the media was harvested and quantified by qPCR; values were normalized to a control plasmid added at the onset of purification. (C) Seven days after induction, intracellular DNA was harvested from the cells and the level of viral DNA relative to host DNA was determined by qPCR. Data are the average of three biological replicates and error bars represent standard deviations of the mean.

BJAB RRV cells were transfected with 2′-*O-*methylated and phosphorothioate-substituted antisense oligonucleotides (ASOs) that target RRV PAN RNA for RNaseH cleavage. 40 h after lytic induction with trichostatin-A (TSA), total RNA was harvested from a subset of the cells and analyzed by Northern blot to confirm knockdown of RRV PAN RNA expression (Fig 1A). Seven days later, the extracellular encapsulated and intracellular DNA were harvested from the remaining cells. Knockdown of RRV PAN RNA modestly reduced (by 40%) the yield of DNase-resistant, encapsulated virus released into the media, as assayed by qPCR of viral DNA (Fig 1B). This phenotype mimics that of a KSHV PAN RNA knockdown [2], but is less severe (only 2-fold), perhaps attributable to less efficient knockdown of RRV compared to KSHV PAN RNA. In contrast, qPCR analysis of intracellular DNA confirms that knockdown of RRV PAN RNA does not affect accumulation of intracellular viral DNA during the lytic phase (Fig 1C). Thus in B-cells, loss of RRV PAN RNA, like that of KSHV, decreases the production of encapsulated viral DNA without affecting the levels of intracellular viral DNA in B-cells.

### KSHV and RRV PAN RNAs are functional homologs

Loss of KSHV and of RRV PAN RNA expression both reduce production of encapsulated progeny virions, despite lacking nucleotide sequence conservation. Therefore, we asked whether one PAN RNA homolog could substitute for the other. Initially, we attempted to transiently express either RRV PAN RNA in KSHV-infected BCBL-1 cells or KSHV PAN RNA in RRV-infected BJAB cells after knockdown of endogenous PAN RNA. However, titrating the amount of transfected rescue plasmid (from 2 to 15 μg) revealed that the reciprocal PAN RNA homolog failed to rescue the loss of released encapsulated virus observed after PAN RNA knockdown (S1A, S1D, S1F and S1I Fig). Yet, levels of intracellular viral DNA replication were unchanged (S1E and S1J Fig). In KSHV-infected BCBL-1 cells, we similarly failed to observe rescue of several late lytic proteins upon transfection of an RRV PAN RNA rescue plasmid (S1A Fig). Using RT-qPCR, we quantified the expression level of each PAN RNA homolog relative to the average expression level of five viral transcripts. Surprisingly, neither PAN RNA was expressed at greater than 1% of the endogenous species-matched PAN RNA in BJAB RRV or BCBL-1 PAN-knockdown cells (S1B and S1G Fig). We conclude that the expression level of transiently transfected PAN RNA is insufficient to rescue the knockdown phenotype in BCBL-1 or BJAB B-cells. Unfortunately, we are unable to confirm this conclusion directly. Unlike the situation where protein-coding genes containing silent codon mutations are made, it is impossible to reintroduce a target RNA in the presence of ASOs without making point mutations in the rescue construct, which may have unanticipated functional effects.

**Fig 2:**
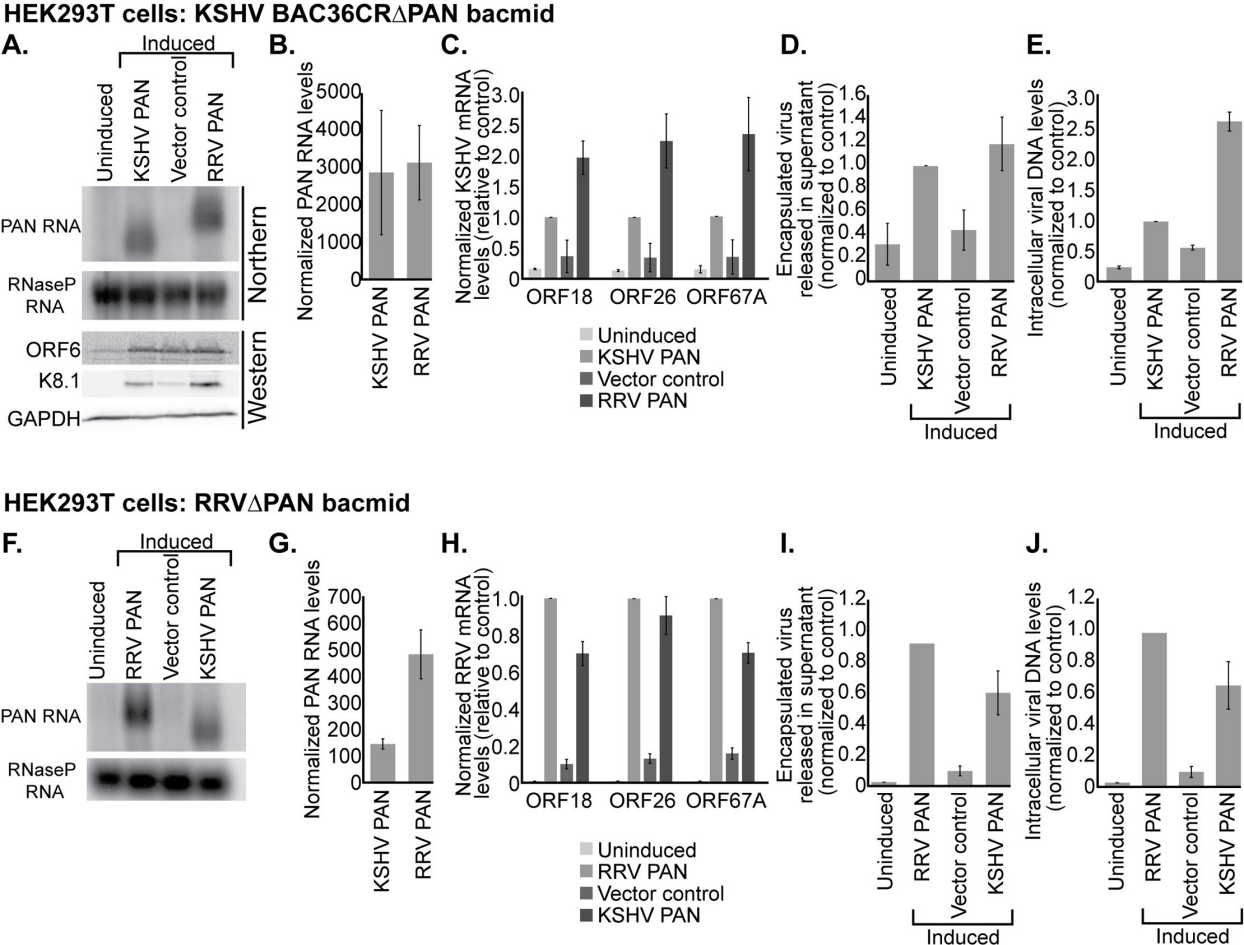
KSHV and RRV PAN RNA are functional homologs. (A) HEK293T cells were transiently transfected with KSHV BAC36CRΔPAN bacmid, kORF50/RTA plasmid and either an empty vector control or a PAN RNA expression vector. 4 h after transfection, the cells were induced into the lytic phase with 600 μM valproic acid. 72 h later, a subset of the cells was harvested for Northern blot analysis of PAN RNA levels and for Western blot analysis of viral protein ORF6 (an early protein) and K8.1 (a late protein). Northern probes complementary to both RRV and KSHV PAN RNA were mixed in the same hybridization reaction. (B) RT-qPCR quantification of PAN RNA levels relative to that of five viral transcripts (see Materials and Methods). (C) RT-qPCR analysis of the early viral transcript ORF18 and two late viral transcripts ORF26 and ORF67A relative to RNaseP RNA. (D) Three days after lytic induction, DNase-resistant encapsulated viral DNA levels in the media were assessed by qPCR and normalized to an external loading control added at the onset of viral DNA isolation. (E) Three days after lytic induction, intracellular DNA was harvested and the level of intracellular viral DNA relative to host DNA was determined by qPCR. The average signal from two primer pairs specific to the viral genome was normalized to the average signal from two primer pairs specific to the human genome. (F) HEK293T cells were transiently transfected with RRVΔPAN bacmid, rORF50/RTA plasmid and either an empty vector control or a PAN RNA expression vector. 4 h after transfection, the cells were induced into the lytic phase with 100 nM TSA. In the same manner as described above, PAN RNA levels (G), viral transcript levels (H), extracellular released viral DNA (I) and intracellular viral DNA (J) were analyzed. Data are the average of three biological replicates and error bars represent standard deviations of the mean.

**Fig 3:**
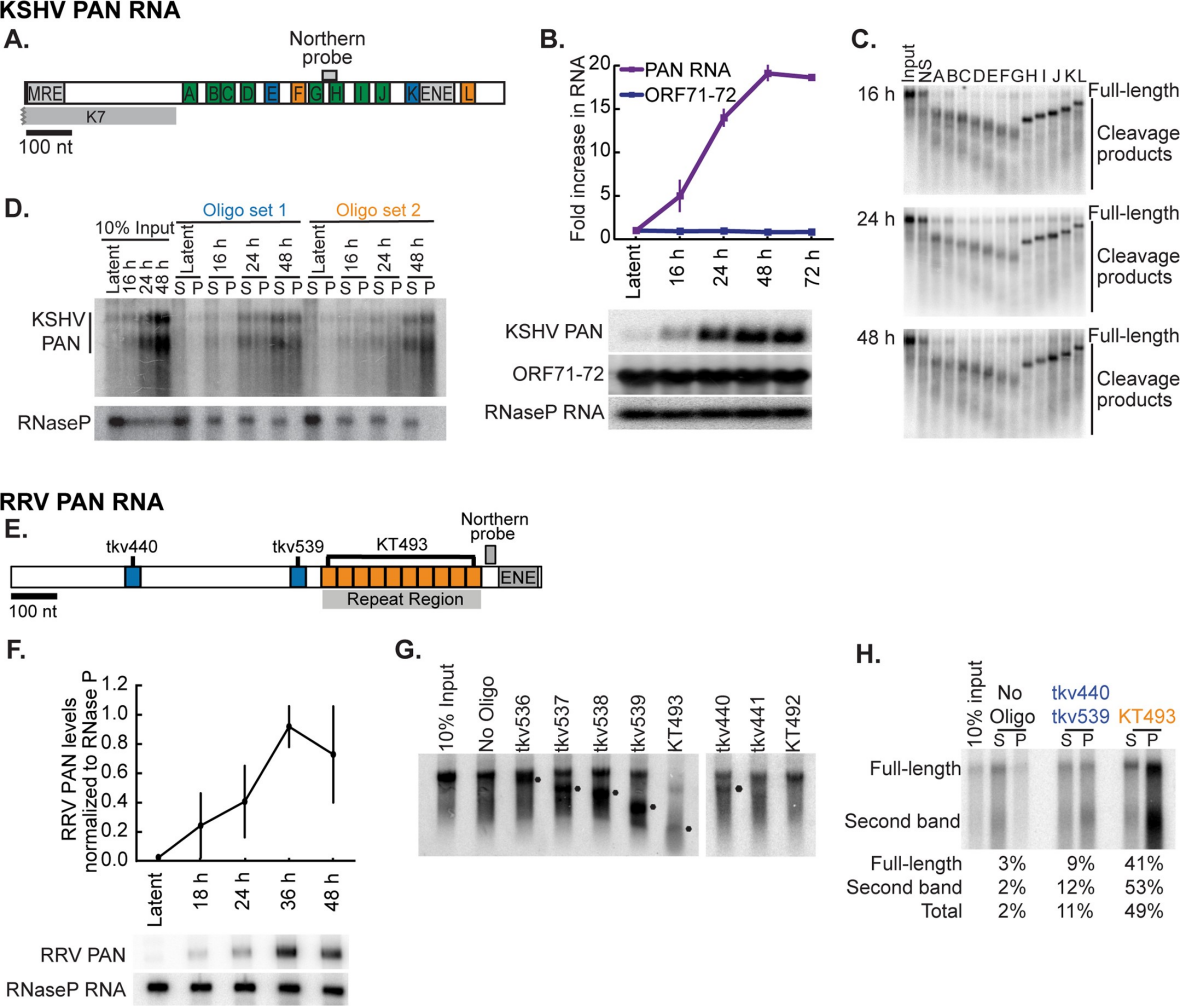
Selection of PAN RNA sequences for use in CHART. (A) Schematic of KSHV PAN RNA indicating the location of known RNA elements MRE (Mta-response element) [57] and ENE (element for nuclear expression) [16], as well as 12 complementary oligonucleotides (A to L) tested for their RNaseH-targeting activity. The overlapping open reading frame K7 is indicated. (B) BCBL-1 cells were induced with 600 μM valproic acid for the indicated time (latent is 0 h); then RNA was harvested. RNA levels of KSHV PAN RNA, KSHV ORF71-72 latent transcript and RNaseP RNA were analyzed by Northern blot. Quantification of three biological replicates normalized to RNase P RNA is shown. (C) Representative RNaseH assays of PAN RNA in BCBL-1 nuclear lysates assessed by Northern blot. No difference in cleavage pattern was observed for the three time points tested. NS: non-specific oligonucleotide complementary to GFP mRNA. (D) Representative enrichment of PAN RNA following CHART RNA isolation with two distinct oligonucleotide sets (blue: oligonucleotides E and K, orange: oligonucleotides F and L). PAN RNA is observed as two bands on a Northern blot following the sonication step of the CHART procedure. Relative to input, approximately 20% of PAN RNA was isolated at each time point. The small fraction of PAN RNA isolated from the latent sample was generated by the 1–3% of cells undergoing spontaneous lytic reactivation. (E) Schematic of RRV PAN RNA indicating the location of the ENE, as well as 3 complementary oligonucleotides tested for their RNaseH-targeting activity. (F) BJAB RRV cells were induced with 500 nM TSA for the indicated time (latent is 0 h); then RNA was harvested. RNA levels of RRV PAN RNA and RNaseP RNA were analyzed by Northern blot. Quantification of three replicates is shown. (G) Representative RNaseH assays of PAN RNA in BCBL-1 nuclear lysates assessed by Northern blot. Cleavage products are indicated by ●. (H) Representative enrichment of RRV PAN RNA following CHART RNA isolation with two distinct oligonucleotide sets (blue: oligonucleotides TKV440 and TKV539, orange: oligonucleotide KT493). PAN RNA is observed as two bands on a Northern blot following the sonication step of the CHART procedure. Quantification of PAN RNA isolated during the CHART procedure is indicated below the Northern blot. S: supernatant P: pellet.

Consequently, we performed rescue experiments with PAN RNA knockout bacmids (Fig 2). We attempted to perform bacmid rescues in several physiologically relevant B-cell lines; however, the low transfection efficiency typical of B-cells in culture prevented expression of sufficient PAN RNA. As a result, knockout bacmid experiments were performed in HEK293T cells, as previously reported [21–23]. A bacmid containing a partial deletion of KSHV PAN RNA (BAC36CRΔPAN) was previously studied and, similar to the results of knockdown experiments, release of encapsulated virus into the media was diminished [10]. In the KSHV genome, 31% of the PAN locus overlaps the K7 open reading frame [24] (Fig 3A), making an exclusive PAN RNA deletion impossible. In contrast, the RRV genome contains no known overlapping open reading frame, allowing a full RRV PAN RNA deletion [25]. To generate an RRVΔPAN bacmid, we eliminated 1300 bp from the wild-type RRV bacmid [26] encompassing the entire RRV PAN RNA transcript, 140 bp of upstream promoter sequence and 22 bp of downstream polyadenylation sequence (S2 Fig). In lieu of the PAN RNA gene, a 1641-bp cassette was inserted that contains a kanamycin/neomycin resistance open reading frame to facilitate bacmid selection during recombineering (S2 Fig).

Next, we tested the ability of KSHV versus RRV PAN RNA to rescue the corresponding PAN RNA deletion bacmids. HEK293T cells were first transiently transfected with KSHV BAC36CRΔPAN bacmid [10], the KSHV lytic transcriptional activator ORF50/RTA and either a KSHV PAN RNA rescue plasmid or a vector control. The vector control showed that the levels of encapsulated viral DNA released into the media were reduced (~40%) and less K8.1 late lytic protein expressed 72 h after lytic induction with 600 μM valproic acid (Fig 2A and 2D). Both phenotypes were rescued not only by expressing KSHV PAN RNA, but also by expressing RRV PAN RNA under the control of the KSHV PAN RNA promoter from a rescue plasmid (Fig 2A and 2D). Using qPCR, we quantified the expression level of each PAN RNA homolog relative to the average expression level of five viral transcripts (Fig 2B), revealing that KSHV and RRV PAN RNAs were each robustly expressed at approximately 2-fold the abundance of PAN RNA in BCBL-1 cells (Figs 2B and S1B). Moreover, in contrast to what is observed in BCBL-1 cells, the levels of intracellular viral DNA replication were reduced in the vector control samples lacking PAN RNA (Fig 2E). This is likely due to the reduced expression of early and late viral mRNAs (Fig 2C), observed previously when the KSHVΔPAN bacmid was introduced into HEK293 cells [10, 11]. Importantly, expression of RRV PAN RNA in the presence of the KSHV BAC36CRΔPAN bacmid rescued late lytic KSHV protein expression and yielded a similar number of extracellular progeny virions, compared to rescue with KSHV PAN RNA (Fig 2C to 2E).

We then performed the converse PAN RNA rescue in the context of the RRVΔPAN genome. HEK293T cells transiently transfected with RRVΔPAN bacmid were induced into the lytic phase by transiently transfecting rORF50/RTA in the presence of 100 nM TSA. The complete knockout of RRV PAN RNA from the bacmid reduced the level of encapsulated virus released into the medium in the presence of a vector control to about 10% relative to a RRV PAN RNA expression vector (Fig 2I). Similar to what was observed for KSHV BAC36CRΔPAN, this phenotype was rescued (6- to 10-fold) by expression of either KSHV or RRV PAN RNA (Fig 2F to 2J). Together the data indicate that KSHV and RRV PAN RNAs, although very different in sequence, can substitute for each other’s function during the lytic phase of herpesviral infection.

### KSHV and RRV PAN RNA do not associate specifically with host or viral chromatin

Previous studies using ChIRP suggested that KSHV PAN RNA associates broadly with host and viral chromatin [10, 11]. We revisited this hypothesis using an alternative chromatin mapping technique known as CHART [20] to identify association sites for KSHV and RRV PAN RNA. Because these two lncRNAs can functionally substitute for one another, we reasoned that any chromatin interaction sites essential for production and release of progeny viral particles should be conserved. To reduce the high background common to both CHART and ChIRP technologies, we applied three strategies: (1) testing both KSHV and RRV PAN RNA homologs for similar sites of chromatin association; (2) exploring PAN RNA association as a function of time after inducing the lytic phase; and (3) using two independent capture oligonucleotide sets for each PAN RNA to limit background and selection of non-specific binding sites.

Expression profiles of PAN RNA during the lytic phase were analyzed after induction of BJAB RRV cells or KSHV-carrying BCBL-1 cells with 500 nM TSA or 600 μM valproic acid, respectively; RNA was collected at multiple time points. For the CHART studies, we assessed PAN RNA-chromatin association when the lncRNAs were at 25%, 75% and 100% of the maximal expression level. Northern blot analysis of KSHV and RRV PAN RNA revealed that these values were achieved at 18, 24 and 48 h for KSHV (Fig 3B) and 16, 24 and 36 h for RRV (Fig 3F).

Candidate CHART capture oligonucleotides were designed according to specifications of the CHART protocol [20] and their ability to bind PAN RNA was determined by RNase H sensitivity assays. RNase H cleaves the RNA strand of RNA-DNA duplexes and thus a DNA oligonucleotide capable of hybridizing to PAN RNA produces a cleavage product that can be detected on a Northern blot. Schematics of the tested CHART PAN RNA oligonucleotides in Fig 3A and 3E illustrate the oligonucleotide binding sites on each of the two PAN RNAs. RRV PAN RNA was only moderately accessible (Fig 3G), while KSHV PAN RNA was fully accessible to antisense oligonucleotide binding and cleavage by RNase H (Fig 3C) at all time points tested. This may reflect a difference in the complement of proteins bound to each PAN RNA.

The pulldown efficiencies of RNase H-candidate CHART capture oligonucleotides were determined by incubating biotin-labeled oligonucleotides with streptavidin beads and nuclear lysate pre-cleared with unbound beads. After stringent high-salt washing, RNAs purified on the streptavidin beads were analyzed by Northern blot (Fig 3D and 3H). To reduce background, two sets of pulldown oligonucleotides were chosen based on the criterion that pulldown efficiency be greater than 10% of the input sample. The exception to the use of two oligonucleotides was the KT493 oligonucleotide, which binds a repeat sequence in RRV PAN RNA and alone has an excellent pulldown efficiency (Fig 3E and 3H, KT493). The second oligonucleotide set for RRV PAN RNA consisted of tkv440 and tkv539. The KSHV CHART oligonucleotide set 1 (oligonucleotides E and K) and set 2 (oligonucleotides F and L) each contained oligonucleotides that target regions near the middle and 3′ end of the transcript, avoiding the ENE stabilization element and not overlapping the K7 transcript (Fig 3A). The pulldown efficiency was ~19% for oligonucleotide set 1 and ~22% for oligonucleotide set 2 (Fig 3D). PAN RNA is observed as two bands on a Northern blot following the sonication step of the CHART procedure. The small fraction of PAN RNA isolated from the latent sample was generated by the 1–3% of cells undergoing spontaneous lytic reactivation.

**Fig 4:**
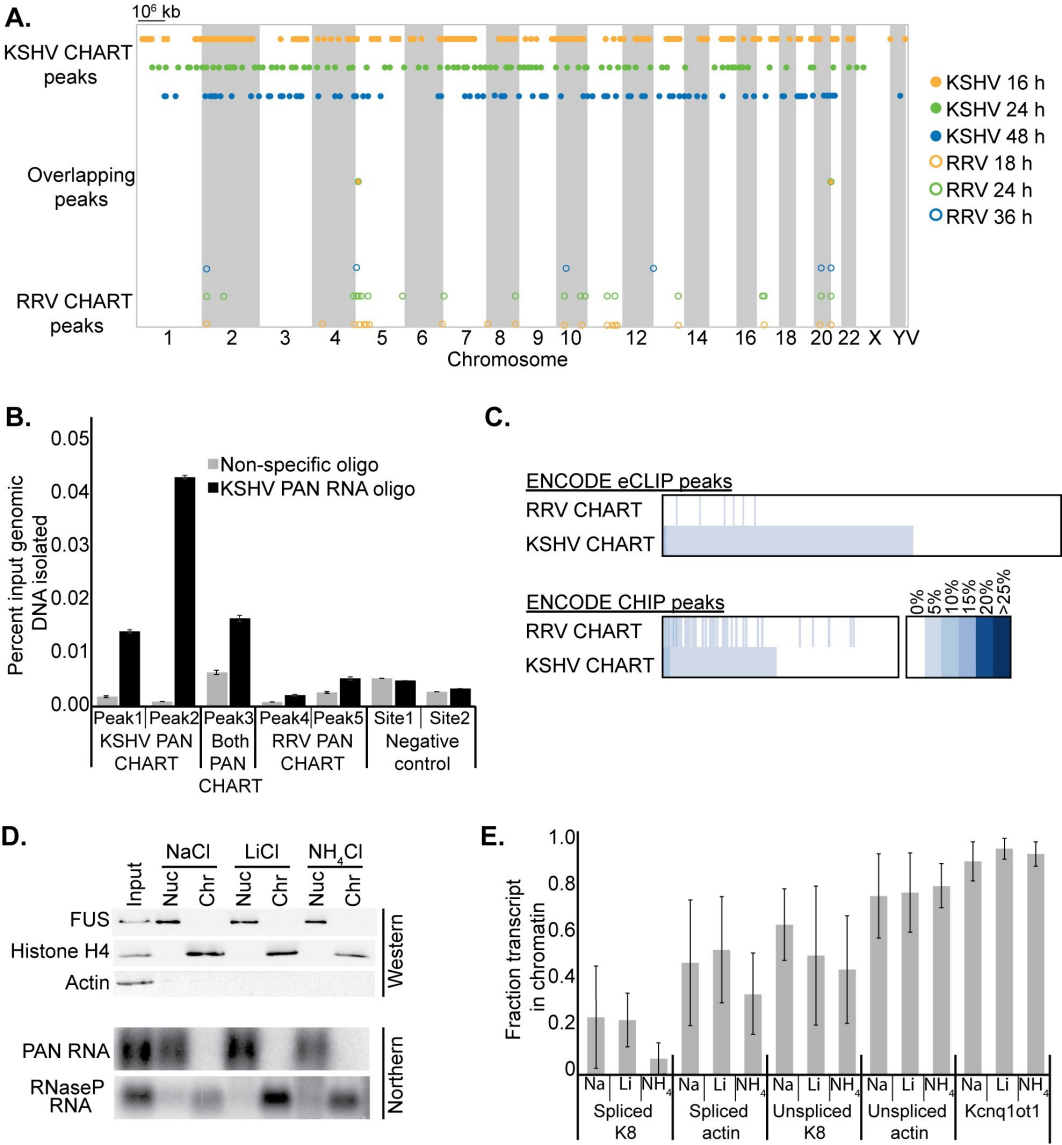
PAN RNA does not associate with chromatin as assessed by CHART or by cell fractionation. (A) Distribution of PAN RNA CHART peak loci along all 23 human chromosomes and on the viral chromosome (V). KSHV CHART peaks are closed circles and RRV CHART peaks are open circles. CHART peaks are plotted for the time point at which the enrichment of that genomic locus was at its maximum. Only two overlapping peaks were called in KSHV and RRV CHART datasets and are plotted on a separate line. See S1 Appendix for detailed data. (B) qPCR validation of KSHV PAN RNA binding to genomic loci identified by CHART peak calling. DNA associated with KSHV PAN RNA was isolated from BCBL-1 cells 48 h after lytic induction using CHART oligonucleotide set 1. See S3 Appendix for qPCR primer and amplicon details. Peaks were identified in only the KSHV dataset (KSHV PAN CHART, Peaks 1 and 2), only the RRV dataset (RRV PAN CHART, Peaks 4 and 5) or both datasets (Both PAN CHART, Peak 4). The negative control sites are on the viral genome and lacked any CHART enrichment in either dataset. (C) Overlap of CHART peaks with ENCODE eCLIP (275 datasets; 104 proteins) and ENCODE CHIP (162 proteins) peaks. Percentages represent the fraction of PAN RNA CHART peaks that overlap each ENCODE dataset. None of the datasets overlap more than 15% of the PAN RNA CHART peaks. The intensity of blue shading represents the extent of overlap between the datasets. Detailed data are shown in S2 Appendix. (D) Subcellular fractionation of lytic BCBL-1 cells does not detect PAN RNA in the chromatin fraction by Northern blot. Western blot for FUS (nucleoplasmic), Histone H4 (chromatin) and GAPDH (cytoplasmic) proteins verifies the purity of the three resulting fractions. Three different salts were exchanged in all fractionation buffers: NaCl, LiCl and NH_4_Cl. (E) qPCR RNA analysis of the fractionated samples, plotted as the amount of each transcript in the chromatin relative to the nucleoplasm. Kcnq1ot1 is a control chromatin-associated ncRNA [28, 29]. Data are the average of three biological replicates.

We evaluated PAN RNA association with chromatin for both KSHV and RRV using two separate antisense capture oligonucleotide sets and several time points representing different levels of PAN RNA expression. BCBL-1 cells containing KSHV and BJAB cells containing RRV were induced into the lytic phase with 600 μM valproic acid or 500 nM TSA, respectively, for up to 48 h prior to performing CHART enrichment of PAN RNA-associated genomic DNA. CHART peaks were called for each time point, relative to input, using MACS2 software (see Materials and Methods). Any genomic locus called as a peak was then assessed for its enrichment score at the other three time points, regardless of whether the MACS2 software independently identified it as a peak in the dataset. Sites of chromatin association at each time point were identified as those genomic DNA loci that associate with PAN RNA in both oligonucleotide sets in at least one of two biological replicates and whose enrichment increased with respect to latent samples, which are largely devoid of PAN RNA (Fig 3B and 3F). PAN RNA CHART peaks were then further separated into three categories: those with an enrichment that peaked at 16, 24 or 48 h for KSHV, and at 18, 24 or 36 h for RRV, respectively. We identified 1034 KSHV and 228 RRV PAN RNA CHART peaks (Fig 4A and S1 Appendix). Only two peaks overlapped between the two PAN RNAs, chr5:1960322–1961985 and chr21:10730325–10731108, albeit with different temporal patterns of enrichment (Fig 4A). Neither of these genomic locations reside within a gene or genomic regulatory feature reported in the UCSC genome annotation.

The lack of overlap between binding sites on host chromatin for the two PAN RNA homologs was not limited by the high-throughput CHART procedure because after isolating genomic DNA associated with KSHV PAN RNA, putative KSHV CHART peaks, but not RRV CHART peaks, could be verified as enriched by qPCR (Fig 4B). Published ChIRP analyses of KSHV PAN RNA identified the viral ORF50 promoter as a specific site of chromatin interaction. Using CHART analysis, we also–to some extent–isolated this region; however, the peaks generated by the two KSHV oligonucleotide sets did not overlap and qPCR of CHART-isolated DNA showed enrichment only with one capture oligonucleotide set (S3 Fig). This suggests that the KSHV ORF50 promoter is likely a region of open chromatin that is readily accessible for non-specific binding events–not a specific site of PAN RNA interaction.

To evaluate whether the same protein factor might bind both PAN RNA homologs, but recruit the lncRNAs to separate, non-overlapping sites on the genome, we compared the PAN RNA CHART peaks to 275 ENCODE eCLIP (enhanced crosslinking and immunoprecipitation) datasets and 162 ENCODE ChIP (chromatin immunoprecipitation) datasets. Of the 276 factors represented in these two analyses, none overlapped with greater than 15% of either the KSHV or RRV CHART peaks (Fig 4C and S2 Appendix). Additionally, no single factor significantly overlapped with both PAN RNA CHART datasets.

Finally, we fractionationated lytic BCBL-1 cells and analyzed the distribution of PAN RNA between the nucleoplasm and chromatin by Northern blot (Fig 4D). Surprisingly, KSHV PAN RNA could not be detected in the chromatin fraction. Three salts (NaCl, LiCl and NH_4_Cl) were used in the same fractionation protocol to minimize the chance that the absence of PAN RNA signal in the chromatin was due to the fractionation buffer. These salts lie on different points along the Hofmeister series–a classification that orders ions by their ability to stabilize and solubilize proteins [27]. The quality of fractionation was verified both by Western blotting and qRT-PCR (Fig 4D and 4E). Although PAN RNA was undetectable in chromatin, unspliced transcripts and the ncRNA Kcnq1ot1 were chromatin-enriched as expected (Fig 4E) [28, 29], indicating that our fractionation protocol is capable of isolating chromatin-associated RNA. These data suggest that the majority of PAN RNA does not associate with chromatin.

### KSHV PAN RNA does not alter the activity of the viral transcription pre-initiation complex

Loss of KSHV PAN RNA results in reduced K8.1 and ORF65 late lytic protein expression ([2], Figs 2 and S1). Transcription of KSHV late lytic genes is facilitated by a viral pre-initiation complex (vPIC) encoded by the viral genome [30]. This six-component complex includes a TATA-binding protein ORF24 and five proteins of unknown function (ORF18, ORF30, ORF31, ORF34, ORF66). Viral genes transcribed by this complex contain a unique TATA box typified by the consensus sequence TATT [31, 32]. PAN RNA does not associate with chromatin and is therefore unlikely to be an integral component of the vPIC. However, post-transcriptional or post-translational modification of vPIC components mediated by PAN RNA could alter the activity of the transcription factor complex.

**Fig 5:**
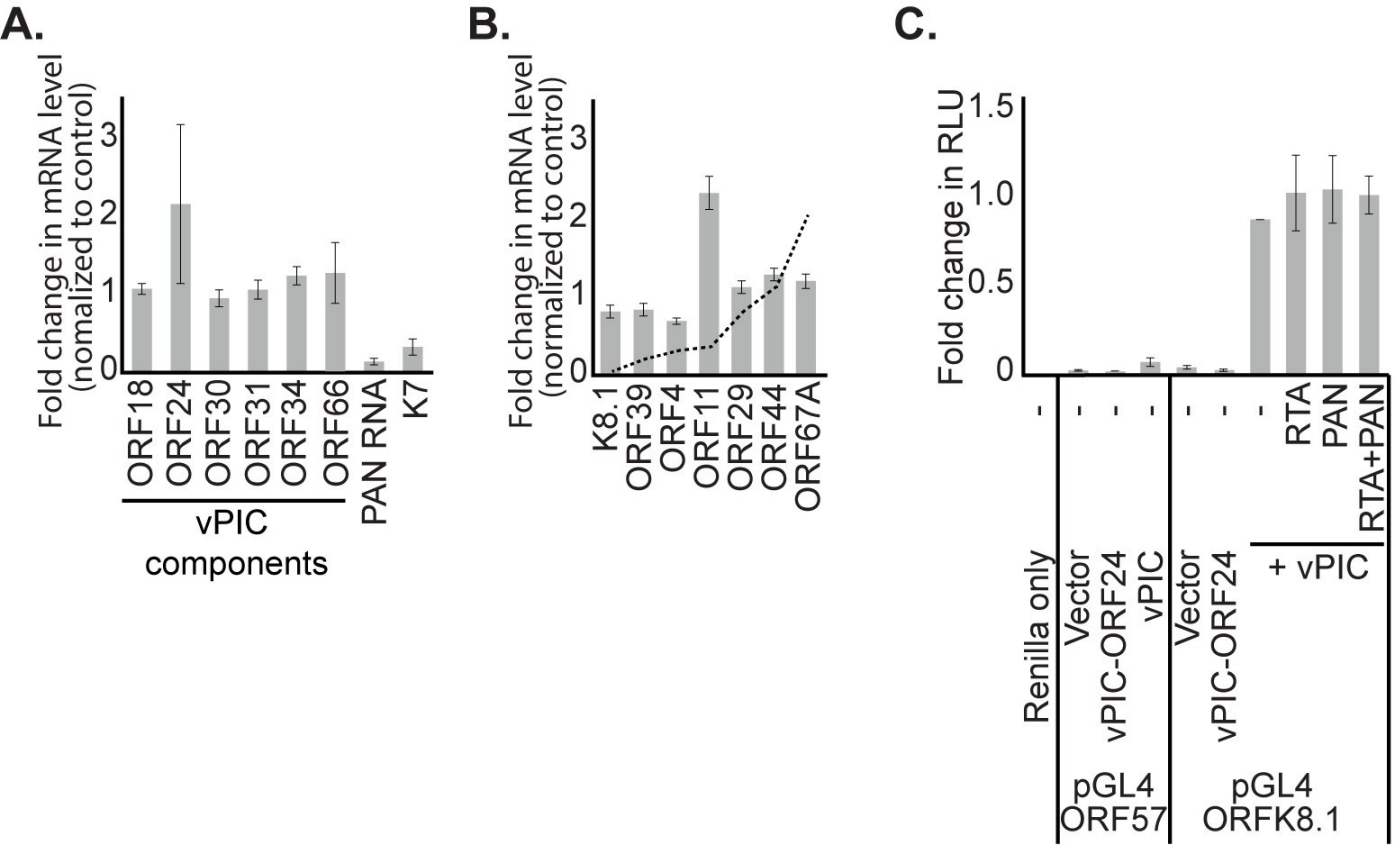
Loss of PAN RNA does not affect the activity of the KSHV late lytic transcription factor complex. BCBL-1 cells were electroporated with oligonucleotides antisense either to GFP mRNA (control KD) or to KSHV PAN RNA and then induced with 600 μM valproic acid for 48 h. RNA was harvested and analyzed by qPCR to determine changes in viral transcription factor mRNA levels (A) and expression of target genes of the viral late lytic transcription factor complex (B). The approximate change in mRNA levels upon knockout of the viral transcription factor ORF31 are shown as a dotted line for reference [33]. Data are the average of three biological replicates. (C) HEK293T cells were transiently transfected with viral late lytic transcription factor expression plasmids as well as luciferase reporter plasmids (pGL4). The firefly luciferase ORF was located downstream of the viral ORF57 promoter (pGL4 ORF57) or the viral K8.1 promoter (pGL4 ORFK8.1). vPIC-ORF24 is a negative control sample that lacks the ORF24 expression plasmid. RTA is a transcription factor that is required for expression of PAN RNA. Samples were normalized to pGL4 ORFK8.1+vPIC+vector. Data represent the average of three biological replicates, each done in three technical replicates.

To test whether KSHV PAN RNA affects vPIC transcriptional activity, we (1) assessed whether knockdown of PAN RNA alters the steady-state mRNA level of any vPIC component; (2) analyzed vPIC-regulated transcript levels upon PAN RNA knockdown; and (3) monitored the influence of PAN RNA expression on a dual luciferase reporter system that expresses firefly luciferase from a vPIC-dependent promoter. At 48 h post lytic induction of BCBL-1 cells, when late lytic transcription is underway, qPCR analyses revealed that the steady-state levels of neither vPIC component mRNAs nor vPIC-regulated transcripts were significantly altered upon knockdown of PAN RNA, as compared to control knockdown samples (Fig 5A and 5B). The approximate fold-change in mRNA levels of viral transcripts upon knockout of the vPIC component, ORF31, are indicated by a dotted line (Fig 5B) [33]. If PAN RNA cooperates with the vPIC to facilitate transcription of the indicated target genes, we would expect a change in the levels of these transcripts to be similar in the absence of PAN RNA to that in the absence of ORF31.

To directly test whether PAN RNA expression affects the activity of the vPIC, we took advantage of a luciferase reporter system whose expression is activated only in the presence of all six viral transcription factors [30]. Upon transient transfection of this vPIC reporter and constructs encoding the six vPIC components in HEK293T cells, a 5-fold increase in luciferase activity was observed when the firefly luciferase gene was present downstream of the late lytic K8.1 promoter, but not downstream of the early ORF57 promoter (Fig 5C). ORF50/RTA expression is required to transcribe PAN RNA from its endogenous promoter [34]. Control samples containing an ORF50/RTA or PAN RNA expression plasmid alone did not affect luciferase levels. When the ORF50/RTA and PAN RNA expression plasmids were both present, and therefore PAN RNA was expressed, the extent of luciferase activation by the vPIC remained unchanged (Fig 5C; RTA+PAN). Although the components of the vPIC complex are conserved in RRV, a late lytic viral transcription factor complex has not yet been described. Together, these data suggest that the reduction in late lytic viral gene expression observed upon PAN RNA knockdown does not occur via the concerted action of this lncRNA and the vPIC.

### PAN RNA is required for efficient nuclear export of transcripts

**Fig 6:**
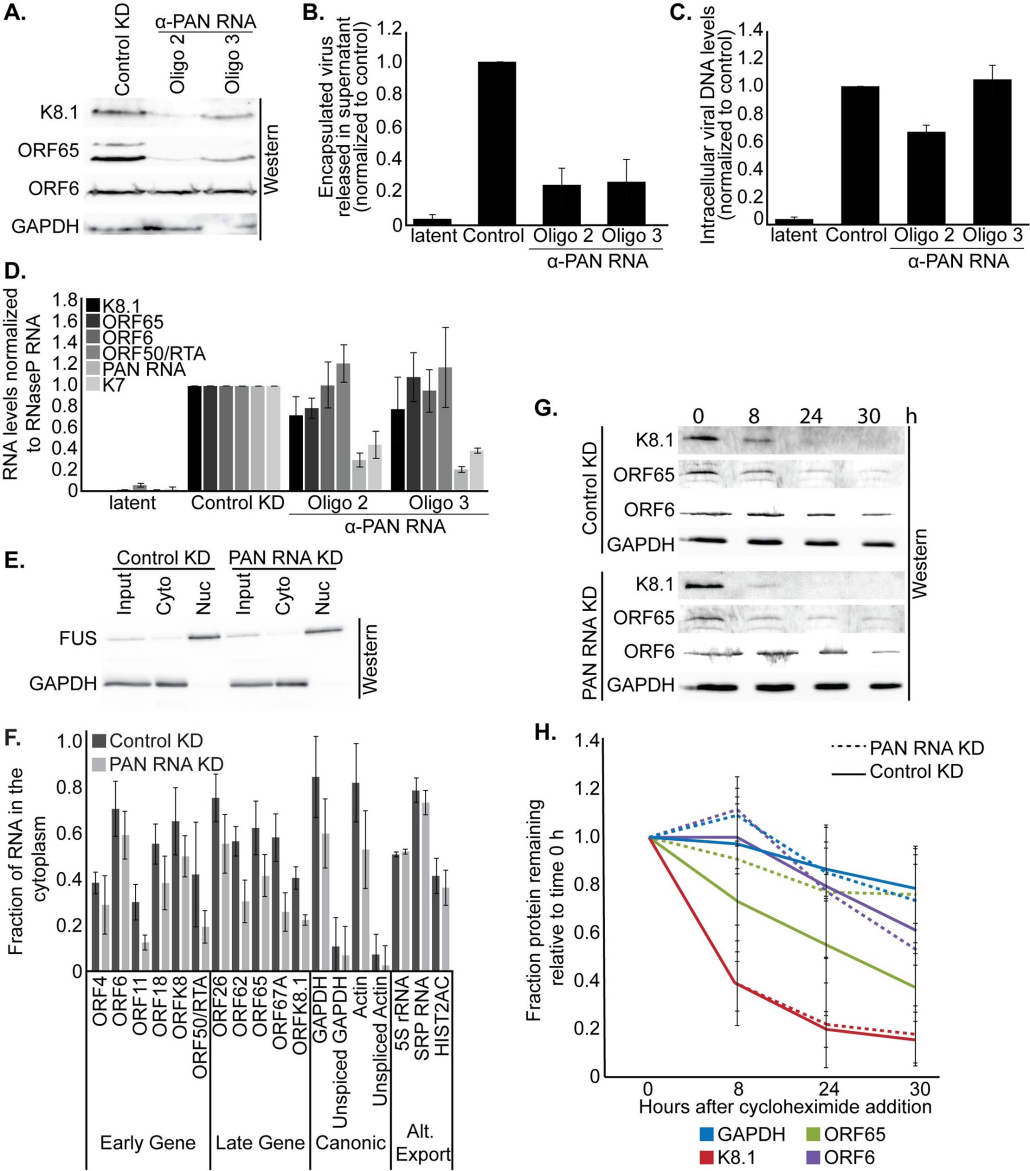
Knockdown of KSHV PAN RNA reduces nuclear export of mRNA transcripts. BCBL-1 TREx cells were electroporated with oligonucleotides antisense either to GFP mRNA (control KD) or to KSHV PAN RNA, followed by induction into the lytic phase with 1 μg/mL doxycycline. (A) 48 h after induction, cells were harvested for Western blot analysis of the early viral protein ORF6, and late viral proteins K8.1 and ORF65. (B) Seven days after induction, encapsulated viral DNA released into the media was harvested and quantified by qPCR; values were normalized to a control plasmid added at the onset of purification. (C) Seven days after induction, intracellular DNA was harvested from the cells and the level of viral DNA relative to host DNA was determined by qPCR. (D) 48 h after induction, a subset of the cells was harvested for RT-qPCR quantification of transcript levels in PAN RNA knockdown cells, relative to those in control knockdown cells. Additional transcript level quantifications are shown in S6 Fig. (E) 24 h after induction, the purity of cellular subfractions was assessed by Western blot for cytoplasmic (GAPDH) and nucleoplasmic (FUS) contents. (F) RT-qPCR analysis of fractionated BCBL-1 TREx cells. The fraction of each transcript present in the cytoplasm, relative to the nucleus, is plotted. (G) Representative Western blot analysis of BCBL-1 TREx cells treated with 100 μg/ml of the translation elongation inhibitor cycloheximide for the indicated times. (H) Quantification of data represented in panel (G). Degradation of the late viral protein ORF65 is slightly reduced following PAN RNA knockdown. Data are the average of three biological replicates and error bars represent standard deviations of the mean.

To determine how loss of PAN RNA results in misregulation of late lytic viral genes, we induced BCBL-1 TREx cells into the lytic phase for 24 h with 1 μg/mL doxycycline following knockdown of PAN RNA or, as a control, GFP mRNA. Doxycycline regulates expression of the inducible gene encoding the lytic activator RTA/ORF50. As observed previously, knockdown of KSHV PAN RNA using two separate antisense oligonucleotides resulted in a specific reduction in late lytic viral protein expression (K8.1 and ORF65) (Fig 6A) and in progeny virions released into the media (Fig 6B), but no change in the intracellular viral genomic DNA copy number (Fig 6C). However, when we isolated total RNA from the same cells and assessed mRNA levels by qRT-PCR, we did not observe a significant reduction in the mRNA encoding late lytic viral proteins upon loss of KSHV PAN RNA expression (Figs 6D and S5). This suggests that the decrease in late lytic protein expression observed upon PAN RNA knockdown is not the result of changes in transcription or RNA degradation rates. Effects on protein levels, without a corresponding change in RNA levels could be a consequence of reduced export of mRNA from the nucleus, reduced protein production, accelerated protein decay or any combination thereof.

PAN RNA is predominantly, if not exclusively nuclear. Therefore direct effects on the cytoplasmic processes of protein production or decay are unlikely. Nonetheless, we assessed whether protein decay rates are altered in the absence of PAN RNA expression. After 48 h of lytic induction, BCBL-1 cells were treated with the translation elongation inhibitor cycloheximide. At 0, 8, 24 and 30 h of translation inhibition, equivalent numbers of cells were harvested and analyzed by Western blot for the early viral protein ORF6, late viral proteins K8.1 and ORF65, as well as the host protein GAPDH (Fig 6G). Little difference in protein decay was observed upon knockdown of PAN RNA, as compared to control knockdown samples (Fig 6H). This suggests that PAN RNA is not influencing protein degradation of late lytic viral proteins.

To evaluate whether PAN RNA affects export of viral mRNA from the nucleus, we fractionated lytically-induced BCBL-1 cells following knockdown of either PAN RNA or, as a control, GFP mRNA. The quality of fractionation was assessed by Western blot and by qRT-PCR (Fig 6E and 6F) for a cytoplasmic marker (GAPDH protein) and nuclear markers (FUS protein, unspliced actin RNA, unspliced GAPDH RNA). The fraction of both host and viral mRNAs decreased in the cytoplasm and increased in the nucleoplasm after knockdown of PAN RNA (Fig 6F). This was true of both late and early lytic viral transcripts, as well as host GAPDH and actin mRNAs. Importantly, transcripts that are exported from the nucleus by alternative pathways, such as 5S rRNA [35, 36], SRP RNA [37] and HIST2AC mRNA [38], are not affected by knockdown of PAN RNA. Fractionation of BJAB-RRV cells, despite evidence for a clean fractionation as assessed by Western blot (S6A Fig), was prone to RNA leakage from isolated nuclei, which complicates analysis of these samples. Nonetheless, a reduction in nuclear mRNA export of viral mRNAs is also observed in BJAB-RRV cells following knockdown of RRV PAN RNA (S6B Fig). We conclude that PAN RNA affects nuclear mRNA export during the lytic phase of viral infection.

## Discussion

We have shown that two herpesviral PAN RNA homologs are functionally interchangeable, despite lacking appreciable nucleotide sequence conservation. Knockdown or knockout of KSHV PAN RNA was previously shown to result in loss of late lytic protein expression, and consequently, a reduction in release of new virions into the surrounding media [2, 10]. In this study, we demonstrate that knockdown or knockout of RRV PAN RNA likewise causes a reduction in release of encapsulated viral DNA (Figs 1 and 2). Furthermore, by expressing either RRV or KSHV PAN RNA from the appropriate herpesvirus species-matched PAN RNA promoter, both PAN RNAs are capable of restoring progeny virion release from HEK293T cells when co-expressed with either a KSHV or RRV PAN RNA knockout bacmid in HEK293T cells (Fig 2). RRV PAN RNA is also capable of rescuing the deficiency in late lytic protein expression observed with the KSHVΔPAN bacmid. In contrast to BCBL-1 and BJAB cells, the level of intracellular viral DNA produced from a PAN RNA knockout bacmid expressed in HEK293T cells is reduced. This suggests that in addition to lacking late lytic viral proteins required for packaging viral DNA, the viral DNA itself is not available for incorporation into progeny virions. This is likely due to a deficiency in robust lytic induction, as reported [10]; we also observed reduced expression of early mRNA, as well as late mRNA (Fig 2). Such subtle differences in phenotype associated with the loss of PAN RNA could be attributable either to cell type differences–a phenomenon that has been observed for other herpesviral gene knockouts and viral gene expression analyses [39–41]–or to the residual level of PAN RNA present after knockdown in B-cells, which may be sufficient to promote robust viral DNA replication.

The herpesviral noncoding PAN RNA is essential to the viral life cycle, but the molecular mechanism by which this lncRNA acts remains unclear. Seven hypothetical models for PAN RNA function have been proposed [42–44]. Three of these posit that PAN RNA associates with chromatin [9–11]. We conclude that the amount of PAN RNA that purportedly associates with chromatin must be very small because we were unable to detect any KSHV PAN RNA in the chromatin following fractionation of BCBL-1 cells, despite detecting other known chromatin-associated RNAs (Fig 4A and 4B). Nonetheless, because KSHV and RRV PAN RNA homologs can functionally substitute for each other, we tested whether direct association with chromatin loci pertinent for producing progeny virions is indeed conserved between these two herpesviruses. We developed a CHART scheme that involved two different capture oligonucleotide sets and three time points during the lytic phase for both herpesviruses (Fig 3). When the CHART peaks representing sites of PAN RNA chromatin association were compared between KSHV and RRV, we observed only two host chromatin loci in common between the two homologs (Fig 4). Neither of these chromatin loci lie within or near annotated genes, chromatin marks or repetitive elements, which makes their relevance questionable. The dearth of peaks that overlap between the KSHV and RRV PAN RNA CHART datasets was confirmed by qPCR analyses demonstrating that KSHV PAN RNA does not associate with chromatin loci identified as RRV CHART peaks (Fig 4B).

We tested whether the two PAN RNA homologs might be localized on separate, non-overlapping regions in the genome either by a DNA binding protein or an RNA binding protein associated with nascent RNA. We compared our PAN RNA CHART peak datasets to all ENCODE CHIP and eCLIP datasets available at the time of our analysis. Of the 264 proteins included in this analysis, none exhibited greater than 15% overlap with either of our datasets. The protein that showed the greatest overlap (14.3%) with the KSHV PAN RNA dataset, but was nearly absent from that of RRV, was FOXK1. The binding site for a different forkhead protein, FOXD3, was enriched in PAN RNA ChIRP peaks [11]. Forkhead transcription factors are the key effectors of many essential signaling pathways [45] and bind the DNA consensus sequence GTAAACA, but flanking sequences or cofactors appear to influence binding of the different family members [46]. However, the correlation of KSHV PAN RNA with forkhead protein binding sites does not appear to be conserved for RRV PAN RNA.

Previous work studying PAN RNA association with chromatin used a technique similar to CHART, known as ChIRP [13]. The ChIRP scheme used would identify any PAN RNA chromatin association sites, but genuine sites might be obscured by the high number of non-specific and off-target background peaks. The raw KSHV PAN RNA ChIRP data [11] are not available for direct comparison with the CHART data presented here, but from the published PAN RNA ChIRP gene list, we do not observe any overlap in chromatin loci between the two datasets. This lack of reproducibility reinforces our conclusion that PAN RNA does not associate with specific sites on host or viral chromatin. The KSHV ORF50 promoter was validated as a site of PAN RNA chromatin association as evidenced by the ability of the demethylases JMJD3 and UTX to interact with the ORF50 promoter only when PAN RNA is expressed [10]; however, we hypothesize that this result is a false positive. The ChIRP study reported that expression of ORF50 is severely reduced in the absence of PAN RNA [10]. The lack of association between PAN RNA-associated factors JMJD3 and UTX and the ORF50 promoter observed on the KSHVΔPAN bacmid could be attributable to reduced ORF50 expression, and hence the extent of open chromatin at this locus. The chromatin sites observed in both the ChIRP and CHART data likely represent non-specific enrichment of open chromatin regions that readily interact with the highly abundant nuclear PAN RNA. This is supported by the poor overlap of PAN RNA CHART peaks between datasets; for each time point, approximately 1% or fewer of the CHART peaks were called in all four datasets (S4 Appendix). At this point we cannot rule out the possibility that PAN RNA globally reorganizes the nucleus through molecular crowding, resulting in occasional, transient, non-specific DNA interactions. It is well established that herpesviruses reorganize the host nucleus and concentrate viral DNA into replication compartments [47]. Together, our results suggest that association of PAN RNA with specific host or viral chromatin loci is neither the primary nor pertinent function of this lncRNA during the viral life cycle.

Campbell and colleagues found that the knockdown of PAN RNA leads to the recruitment of LANA protein at viral promoters, an activity normally associated with latency, suggesting a role for PAN RNA in latent-lytic transitions and chromatin association [12]. However, our study indicates that PAN RNA does not associate with the viral chromatin above background levels. We interpret the effect of PAN RNA on LANA, despite *in vitro* binding studies, to be due to inhibition of virion production. The unpackaged state of newly synthesized viral genomes in the absence of PAN RNA may lead to increased accessibility for LANA binding.

Although PAN RNA is considered exclusively nuclear, ribosome footprint profiling detected ribosome-protected regions of KSHV PAN RNA within three open reading frames (ORFs) at the 5′ end of the transcript, which overlaps with K7 [48]. Due to the extreme abundance of PAN RNA, if less than 1% of the PAN RNA transcripts escape the nucleus and are translated, a non-trivial amount of PAN protein could be produced [42]. Comparison of the sequence of KSHV and RRV PAN RNAs indicate that the ORFs are not conserved in nucleotide sequence, peptide sequence or peptide length (S4A and S4B Fig). KSHV PAN ORF1.1 contains a putative signal peptide, which might permit the peptide to traverse the secretory pathway [48]. A peptide of different length and sequence could be expressed from RRV PAN RNA (nts 682–786) that likewise contains a putative signal peptide (S4C Fig). The coding potential of PAN RNA homologs remains a topic for further study.

We suggest that the absence of PAN RNA during the viral lytic phase perturbs efficient nuclear export of mRNAs. A modest reduction in nuclear export of several viral and host mRNAs was observed when either KSHV or RRV PAN RNA was eliminated indicating that this is a conserved phenotype of PAN RNAs. Despite the change in the nuclear-cytoplasmic distribution of early viral transcripts, and host GAPDH and actin mRNA, we did not observe a change in corresponding protein levels. A reduction in mRNA export likely has a more pronounced effect on diminishing the level of late lytic viral proteins due to the absence of late lytic transcription and translation prior to PAN RNA expression. In contrast, expression of host transcripts and early viral transcripts may generate a protein population that remains unchanged for the duration of the PAN RNA knockdown experiments. Consistent with this hypothesis, the half-life of GAPDH has been estimated to be ~38 h–significantly longer than the time frame of our assays [49]. Whether PAN RNA directly interfaces with mRNA nuclear export machinery and why late lytic protein expression, specifically, is affected remains a topic of future research.

We hypothesize that PAN RNA’s function is to associate with RNA binding proteins, such as PABPC [50], that are relocalized from the cytoplasm to the nucleus during viral infection. In the absence of PAN RNA, such as in the case of a knockout or knockdown, an abundance of unsequestered RNA binding proteins mislocalized in the nucleus could interfere with nuclear mRNA export, viral replication and gene expression. A study of PAN RNA secondary structure in the cell indicates that some regions of KSHV PAN RNA are protected from chemical probing and may be sites of protein interaction [44].

PAN RNA represents a cautionary tale for the investigation of RNA association with chromatin, whereby sequence reads of DNA cross-linked to an abundant RNA give the appearance of specific interactions. Rather than the latest technological advances, well-controlled, unbiased approaches should be applied to determine the true biological role of the ncRNA under investigation. Protein-RNA co-purification studies may also be subject to false positive interactions. This has been especially true of PAN RNA, which–by virtue of its abundance and diffuse nuclear distribution–can transiently and non-specifically associate with many factors.

Molecular tools available for the study of lncRNAs such as PAN RNA are still under development. Most lncRNAs are of low abundance and lack obvious nucleotide sequence conservation that aids in identification of essential sequence elements. Here, we have exploited the use of a ncRNA from a related virus to provide a genetically-tractable model system. Viruses thus offer a unique advantage over host ncRNAs for interrogating questions in RNA biology. PAN RNAs may be multifunctional and fulfill different roles at each stage of the lytic phase. Moving forward, carefully designed and innovative approaches are needed to expand insights into the multifaceted functions of gammaherpesviral PAN RNAs.

## Materials and methods

### Cell culture, inductions and antibodies

Body cavity based lymphoma-1 (BCBL-1) [51], BCBL-1 TREx-RTA (gift from Jae Jung, USC) and BJAB RRV cells [19] were maintained in RPMI supplemented with 1% L-glutamine and 20% FBS. Human embryonic kidney 293T (HEK293T) cells (ATCC CRL-3216) were maintained in DMEM supplemented with 1% L-glutamine and 10% FBS. BCBL-1 cells were induced with 600 μM valproic acid, BCBL-1 TREx-RTA were induced with 1.5 μg/mL doxycycline, BJAB RRV cells were induced with 100 or 500 nM TSA, where indicated. For transfection of BCBL-1 and BCBL-1 TREx-RTA cells, 10 million cells were pelleted and washed once with media lacking serum. Either 2 nmoles of RNaseH targeting oligonucleotide or 15 μg of plasmid DNA were electroporated into 10 million cells in a 0.4 cm cuvette at 975 μF/210 mV. HEK293T cells were transfected with Mirus TransIT-293 reagent according to the manufacturer’s directions. Antibodies used are 1:1000 anti-FUS (Proteintech Group, 11570-1-AP), 1:1000 anti-Histone H4 (Upstate Cell Signaling Solutions, 07–108), 1:2000 anti-GAPDH (Sigma G8795), 1:500 anti-KSHV K8.1 (Advanced Biochemicals Incorporated) and 1:1000 anti-KSHV ORF6 (gift from G. Hayward at The Johns Hopkins University). Cells were treated with 100 μg/mL cycloheximide for the indicated times.

### Cloning and plasmids

The RRV wild-type bacmid was a kind gift from the Desrosiers lab (University of Miami) [26]. This bacmid was used by Genebridges to construct the RRVΔPAN bacmid, which was confirmed by direct sequencing and enzymatic digestion (S2B Fig). Luciferase reporters and vPIC transcription factor expression plasmids were previously described [30]. KSHV PAN RNA and RRV PAN RNA fragments were assembled downstream of the KSHV PAN promoter using Gibson PCR assembly and cloned into the BamHI and SpeI sites of p4030-16TR [52]. KSHV PAN RNA was cloned downstream of the RRV PAN promoter using Gibson PCR assembly into pCDEF4-RRV PAN RNA [8].

### RNaseH assays

Endogenous RNaseH cleavage was assayed as described [53]. Briefly, 1–5 x 10^7^ cells were resuspended in 100 μL/10^7^ cells of Sucrose Buffer I (0.32 M sucrose, 3 mM CaCl_2_, 2 mM MgAc_2_, 0.1 mM EDTA, 10 mM Tris-HCl pH 8.0, 1 mM DTT, 0.5 mM PMSF and 0.5% (v/v) NP-40). After centrifuging the lysate at 500 x g for 5 min at 4°C, the supernatant was removed. Nuclei were washed in 1 mL Sucrose Buffer I lacking NP-40. After removing the supernatant, the nuclei pellet was resuspended in 20 μL/10^7^ cells of Low Salt RNA Buffer (20 mM HEPES, pH 7.9, 25% glycerol, 1.5 mM MgCl_2_, 0.02 M KCl, 0.2 mM EDTA, 0.5 mM DTT, 0.5 mM PMSF). 1/5 volume of High Salt RNA Buffer (20 mM HEPES, pH 7.9, 25% glycerol, 1.5 mM MgCl_2_, 0.8 M KCl, 0.2 mM EDTA, 0.5 mM DTT, 0.5 mM PMSF, 1% NP-40) was added five times. The nuclear extract was then incubated at 4°C on a rotary platform for 20 min and diluted 1:2.5 with RNA Nuclear Diluent (25 mM HEPES pH 7.6, 25% glycerol, 0.1 mM EDTA, 0.5 mM DTT, 0.5 mM PMSF). 10 μL 100 μM DNA oligonucleotides were added to 90 μL of nuclear extract and incubated for 30 min at 37°C. Reactions were stopped by adding 0.5 mL TRIzol and purified according to the manufacturer’s protocol. Samples were run on a 1.2% formaldehyde agarose for a northern blot analysis.

### CHART assay

CHART was carried out as described [20]. CHART capture oligonucleotides were designed according to instructions in [20]. The pulldown oligonucleotides consisted of OLIGO SEQ-3′ end-TEG and were ordered from IDT. Sequencing libraries were constructed by the Yale Center for Genomic Analysis (YCGA) and Yale Stem Cell Genomics Core using Illumina CHIP-Seq Sample Prep Kit. Sequencing was performed on Illumina HISeq 2500 or 2000 instruments. Each CHART time point was prepared in two biological replicates. CHART-seq data were deposited in the Gene Expression Omnibus (GEO) under the accession number GSE121268.

### Bioinformatic analyses

50-bp deep sequencing reads were mapped using Bowtie2 default settings to the custom indices containing both the appropriate viral genome (RRV accession #AF210726; KSHV accession #GQ994935) and the human genome. Reads that mapped to multiple genomic loci were removed using samtools (samtools view input.bam | grep AS:i:0 | grep -v XS:i:0). MACS2 (https://pypi.python.org/pypi/MACS2) [54] was used to call peaks and determine the fold enrichment for each dataset. For each time point, peaks were required to be (1) called by the MACS2 software in at least one biological replicate for both CHART capture oligonucleotide datasets, (2) have an enrichment score at least 1.5-fold greater than the latent sample, and (3) be absent from the no-oligo control pulldown. For each peak that met these criteria, the average MACS2 enrichment score at that locus was determined for both biological replicates for each of the three lytic phase time points. Each CHART peak was categorized by the time point at which this average enrichment score was highest. CHART peaks from KSHV and RRV were designated as overlapping if the peak regions called by MACS2 overlapped by at least 100 bp. ENCODE [55] CHIP and CLIP datasets were downloaded from www.encodeproject.org and compared to the KSHV and RRV peak genomic loci using the BEDTools suite intersect sub-command [56].

### Cell fractionation

Fractionation was performed on ice with pre-chilled buffers using either 36 h-induced KSHV-infected BCBL-1 cells or 43 h-induced BJAB-RRV cells. The washed and pelleted cells were resuspended in 100 μL of RLB buffer (10 mM Tris pH 7.5, 140 mM NaCl, 1.5 mM MgCl_2,_ 10 mM beta-glycerophosphate, 0.5% Nonidet P-40). Digitonin (Sigma, D-1407) was added to a final concentration of 25 μg/mL while wheat germ agglutinin (Sigma, L9640) was added to a final concentration of 1 μg/mL (BCBL-1) or 1 mg/mL (BJAB-RRV) and the cells were incubated on ice for 5 min to allow cell permeation. For lysis, the cells were carefully layered over 300 μL of RLB buffer containing sucrose (10 mM Tris pH 7.5, 140 mM NaCl, 1.5 mM MgCl_2_, 24% (wt/vol) sucrose, 10 mM beta-glycerophosphate, 0.5% NP-40) and then centrifuged at 13,000 x g for 10 min at 4°C. After centrifugation, supernatants were transferred into a fresh microcentrifuge tube and designated as cytoplasmic fractions. The nuclear pellets were washed three times in 100 μL of RLB buffer and spun down at 400 x g for 4 min at 4°C; the first of which contained digitonin at a final concentration of 25 μg/mL. The pelleted nuclei were resuspended in 30 μL of NUN1 buffer (20 mM Tris pH 7.9, 75 mM NaCl, 0.5 mM EDTA, 0.125 mM PMSF, 50% glycerol, 10 mM beta-glycerophosphate, 0.1 mg/ml tRNA, 1x protease inhibitor) and incubated on ice for 5 min. The nuclei were then lysed by adding 300 μL of NUN2 buffer (20 mM Hepes pH 7.6, 7.5 mM MgCl2, 0.2 mM EDTA, 0.1 mg/ml tRNA, 0.3 M NaCl, 1 M urea, 10 mM beta-glycerophosphate, 1% Nonidet P-40, 1 mM DTT, 1x protease inhibitor). After incubation on ice for 15 min with occasional vortexing and centrifugation at 15,000 x g for 15 min at 4°C, the supernatants were transferred to fresh microcentrifuge tubes as the nucleoplasmic fractions. The chromatin-associated pellets were washed three times with 50 μL of NUN2 buffer, spun at 15,000 x g for 4 min at 4°C, and 100 μL of chromatin buffer (50 mM HEPES pH 7.4, 100 mM NaCl, 0.1% SDS, 0.5% sodium deoxycholate, 1% NP-40, 1 mM DTT, 10 mM beta-glycerophosphate, 1x protease inhibitor) added. The chromatin-associated pellet was then sonicated at 4°C for 10 cycles of 30 sec on, 30 sec off, using a Diagenode Bioruptor Pico sonication device. Input samples were sonicated using the same conditions. NaCl was replaced with LiCl or NH_4_Cl in all buffers for indicated fractionations. One half of each fraction was prepared for RNA and protein analysis. RNA was extracted with TRIzol per the manufacturer’s instructions. The purity of the resulting fractions was assessed by qPCR and Western blot.

### vPIC luciferase assay

9 x 10^4^ HEK293T cells were seeded per well in a 12-well plate. Transfections were conducted using Mirus TransIT-293 transfection reagent with OPTI-MEM media 24 h post plating. pGL4.16 constructs (pGL4.16 ORF57 and pGL4.16 K8.1; [30]) and a *Renilla* control vector were used at a 1:1 molar ratio (300 ng /sample). Total DNA was kept constant in each sample by adding pBluescript SK+. Viral transcription factor plasmids were transfected in equimolar amounts. Dual luciferase assays were conducted 24 h post transfection with the Dual-Luciferase Reporter Assay System (Promega, #E1910). The reagents were prepared according to the manufacturer’s instructions and measurements were conducted with a GloMax-Multi Detection System (Promega). Luciferase measurements were recorded as the ratio of firefly to *Renilla* luciferase activity and normalized to sample 7 (pGL4.16 K8.1 vPIC). Data presented were from four independent experiments, each using mean values of technical triplicate samples.

### RT-qPCR analysis

RNA was purified with TRIzol and treated with RQ1 DNase (Promega) according to the manufacturer’s protocols. 1 μg of RNA was used to generate cDNA with random hexamer primers and Superscript III (Invitrogen) using the recommended protocol. cDNA was diluted 3-fold and 0.75 μL was analyzed in a 15-μL qPCR reaction using FastStart Essential DNA Green Master (Roche) SYBR reagent on a Roche Lightcycler 96. RNA levels were normalized to RNaseP RNA levels, which do not change during lytic induction. To determine the extent of PAN RNA overexpression in HEK293T cells relative to BCBL-1 cells, PAN RNA levels were normalized to the average CT value of five viral transcripts (ORF18, ORF26, ORF4, ORF62 and ORF67A), which accounts for variances in viral genome copy number and induction efficiency between samples. For samples obtained from the same cell line, the fold-change in PAN RNA levels calculated using RNaseP RNA was comparable to the calculation using the five viral transcripts.

### Analysis of intracellular viral DNA levels

Seven days after lytic phase induction, 3 million cells were pelleted, washed with PBS and resuspended in genomic DNA isolation buffer (100 mM NaCl, 10 mM Tris-Cl pH 8, 25 mM EDTA, 0.5% SDS) with 0.1 mg/mL proteinase K. The solution was incubated overnight at 40^°^C, phenol extracted, ethanol precipitated and diluted to 30 ng/μL prior to analysis by qPCR. The average signal from two primer pairs specific to the viral genome was normalized to the average signal from two primer pairs specific to the human genome.

### Analysis of supernatant viral levels

Seven days after lytic phase induction, 1.5 mL of supernatant was collected, passed through a 0.45 micron filter and incubated with 20 units/mL DNase One (New England Biolabs) for 1 h at 37^°^C. Proteinase K lysis buffer (0.75% SDS, 0.1 M NaCl; 50 mM Tris, pH 7.5; 10 mM EDTA, 0.1 mg/mL proteinase K) was added to a final volume of 2 mL and then incubated at 40^°^C for 1 h. 1 ng/mL of a control plasmid (psiCHECK-2) was added to each sample as a normalization control for loss of DNA during subsequent phenol chloroform extraction and ethanol extraction. DNA was resuspended in 15 μL of ddH_2_O (resulting in 100-fold concentration) and analyzed by qPCR. The average signal from two primer pairs specific to the viral genome was normalized to the signal from the control plasmid.

## Supporting information

S1 Appendix PAN RNA CHART peak coordinates. (XLSX)

S2 Appendix Comparison of ENCODE and CHART datasets. (XLSX)

S3 Appendix Oligonucleotide sequences. (XLSX)

S4 Appendix Reproducibility of PAN RNA CHART peak calling between replicates. (XLSX)

S1 Fig Attempted rescue of PAN RNA knockdown in BCBL-1 and BJAB RRV cells. (A) BCBL-1 TREx cells were electroporated with oligonucleotides antisense either to GFP mRNA (control KD) or to KSHV PAN RNA, as well as increasing amounts of plasmid encoding RRV PAN RNA under the control of the KSHV PAN RNA promoter. The total DNA concentrations were kept constant by adding empty pBluescript vector. Following electroporation, cells were induced into the lytic phase with 1.5 μg/mL doxycycline. 48 h after induction, a subset of the cells was harvested for Northern blot analysis of PAN RNA levels and for Western blot analysis of late lytic proteins K8.1 and ORF65 and early protein ORF6. (B) RT-qPCR quantification of PAN RNA levels relative to the average of five viral transcripts (ORF18, ORF26, ORF4, ORF62 and ORF67A). RRV PAN represents data from KSHV PAN RNA KD with 15 μg RRV PAN RNA expression vector. (C) RT-qPCR analysis of the early viral transcript ORF18 and two late viral transcripts ORF26 and ORF67A. (D) Seven days after lytic induction, DNase-resistant encapsulated viral DNA levels in the media were assessed by qPCR and normalized to an external loading control added at the onset of viral DNA isolation. (E) Seven days after lytic induction, intracellular DNA was harvested and the level of intracellular viral DNA relative to host DNA was determined by qPCR. The average signal from two primer pairs specific to the viral genome was normalized to the average signal from two primer pairs specific to the human genome. (F) BJAB RRV cells were electroporated with oligonucleotides antisense either to GFP mRNA (control KD) or to RRV PAN RNA, as well as increasing amounts of plasmid encoding KSHV PAN RNA under the control of the RRV PAN RNA promoter. The total DNA concentration was kept constant by adding empty pBluescript vector. Following electroporation, cells were induced into the lytic phase with 100 nM TSA. 40 h after induction, a subset of the cells was harvested for Northern blot analysis of PAN RNA levels and for Western blot analysis of late lytic protein expression. In the same manner as described above, PAN RNAs levels (G), viral transcript levels (H), extracellular released viral DNA (I) and intracellular viral DNA (J) were analyzed. Data are the average of at least two biological replicates; error bars represent standard deviations of the mean.
(TIF)

S2 Fig Characterization of RRVΔPAN bacmid. (A) Sequence of the DNA cassette inserted at the RRV PAN RNA locus. The entire PAN RNA sequence was deleted, including 140 bps upstream and 22 bp downstream that are necessary to express RRV PAN RNA [8]. A 1641-bp cassette was inserted between nucleotides 22394 and 23693 of the RRV genome reference sequence (accession number AF210726). Lower case: wild-type RRV bacmid sequence. Upper case: inserted DNA sequence including the PGK promoter (purple), kanamycin/neomycin resistance open reading frame (grey) and two FRT sites (green). (B) Ethidium bromide stained agarose gel of the RRV bacmid digested with indicated restriction enzymes. These analyses and sequencing revealed no apparent rearrangements between the wild-type (WT) and **Δ**PAN RRV bacmids.
(TIF)

S3 Fig PAN RNA CHART analysis fails to reproduce enrichment of the KSHV ORF50 promoter determined by ChIRP. (A) qPCR of DNA isolated by KSHV PAN RNA CHART oligonucleotide set 1 (see Fig 3A and 3D) using published primers for the KSHV ORF50 promoter region [10]. (B) Genome browser view of the KSHV genome displaying KSHV CHART data from the region of the ORF50 promoter. qPCR primers overlapping the previously reported ChIRP enriched region [10] are shown in yellow. Set 1 (blue) and Set 2 (green) represent the KSHV PAN RNA CHART capture oligonucleotide sets. Mock denotes the sequencing data from a control CHART enrichment lacking a capture oligonucleotide. Sites of enriched DNA from the two KSHV CHART oligonucleotide sets do not overlap.
(TIF)

S4 Fig KSHV PAN RNA short open reading frames are not conserved in RRV PAN RNA. (A) Sequence alignment of a portion of the KSHV and RRV PAN loci. Candidate KSHV open reading frames identified by ribosome footprint profiling [48] are indicated (ORF1.1, ORF1.2 and ORF1.3). Two putative overlapping RRV open reading frames are indicated (ORF1 and ORF3). (B) Translation of indicated open reading frames. The peptide sequences are not conserved between viruses. (C) Peptide sequence of an open reading frame identified at residues 682–786 of RRV PAN RNA. This sequence is predicted to contain a secretory signal peptide by the SignalP 4.1 prediction program (www.cbs.dtu.dk/services/SignalP/). C-score predicts cleavage site, S-score predicts signal peptide location, Y-score is a combined cleavage site score that accounts for the location of the signal peptide.
(TIF)

S5 Fig Knockdown of PAN RNA in BCBL-1 cells does not affect steady-state transcript levels. BCBL-1 TREx cells were electroporated with oligonucleotides antisense either to GFP mRNA (control KD) or to KSHV PAN RNA and then induced into the lytic phase with 1 μg/mL doxycycline. 48 h after induction, a subset of the cells was harvested for RT-qPCR quantification of transcript levels in PAN RNA knockdown cells, relative to those in control knockdown cells. The levels of KSHV ORF11 mRNA, and host unspliced GAPDH and unspliced actin transcripts increased upon PAN RNA knockdown.
(TIF)

S6 Fig Knockdown of RRV PAN RNA modestly reduces nuclear export of viral mRNA transcripts. BJAB RRV cells were induced with 100 nM trichostatin-A (TSA) following electroporation with antisense oligonucleotides complementary to either GFP mRNA (control KD) or RRV PAN RNA (Oligo 493). After 40 h of lytic induction, cytoplasmic and nuclear fractions were isolated. (A) Western blot analysis assesses the purity of the cytoplasmic (GAPDH) and nuclear (FUS) fractions. (B) RT-qPCR analysis of fractionated BJAB-RRV cells. The fraction of each transcript present in the cytoplasm, relative to the nucleus is plotted. Despite extensive optimization of conditions, nuclei isolated from BJAB-RRV cells were prone to RNA leakage, which obscures the results of this analysis. Unspliced actin did not yield a signal above background samples lacking reverse transcription.
(TIF)
