# Supplementary figures and images for: Two herpesviral noncoding PAN RNAs are functionally homologous but do not associate with common chromatin loci
Source: PLoS Pathog. 2018 Nov 1;14(11):e1007389. doi: 10.1371/journal.ppat.1007389 (PMC6233925; doi:10.1371/journal.ppat.1007389)

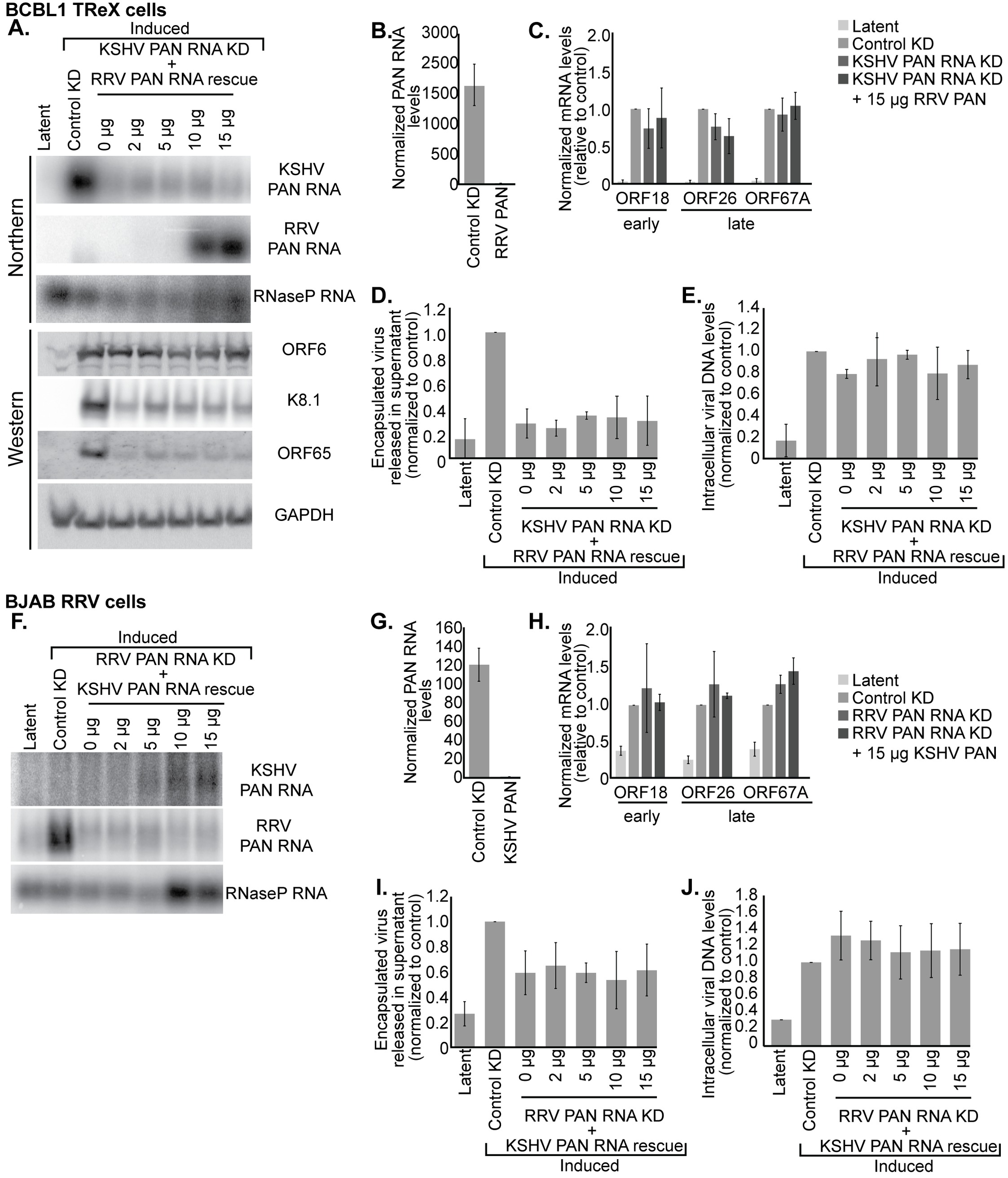

Supplement: S1 Fig — (A) BCBL-1 TREx cells were electroporated with oligonucleotides antisense either to GFP mRNA (control KD) or to KSHV PAN RNA, as well as increasing amounts of plasmid encoding RRV PAN RNA under the control of the KSHV PAN RNA promoter. The total DNA concentrations were kept constant by adding empty pBluescript vector. Following electroporation, cells were induced into the lytic phase with 1.5 μg/mL doxycycline. 48 h after induction, a subset of the cells was harvested for Northern blot analysis of PAN RNA levels and for Western blot analysis of late lytic proteins K8.1 and ORF65 and early protein ORF6. (B) RT-qPCR quantification of PAN RNA levels relative to the average of five viral transcripts (ORF18, ORF26, ORF4, ORF62 and ORF67A). RRV PAN represents data from KSHV PAN RNA KD with 15 μg RRV PAN RNA expression vector. (C) RT-qPCR analysis of the early viral transcript ORF18 and two late viral transcripts ORF26 and ORF67A. (D) Seven days after lytic induction, DNase-resistant encapsulated viral DNA levels in the media were assessed by qPCR and normalized to an external loading control added at the onset of viral DNA isolation. (E) Seven days after lytic induction, intracellular DNA was harvested and the level of intracellular viral DNA relative to host DNA was determined by qPCR. The average signal from two primer pairs specific to the viral genome was normalized to the average signal from two primer pairs specific to the human genome. (F) BJAB RRV cells were electroporated with oligonucleotides antisense either to GFP mRNA (control KD) or to RRV PAN RNA, as well as increasing amounts of plasmid encoding KSHV PAN RNA under the control of the RRV PAN RNA promoter. The total DNA concentration was kept constant by adding empty pBluescript vector. Following electroporation, cells were induced into the lytic phase with 100 nM TSA. 40 h after induction, a subset of the cells was harvested for Northern blot analysis of PAN RNA levels and for Western blot analysis of late [file ppat.1007389.s005.tif]

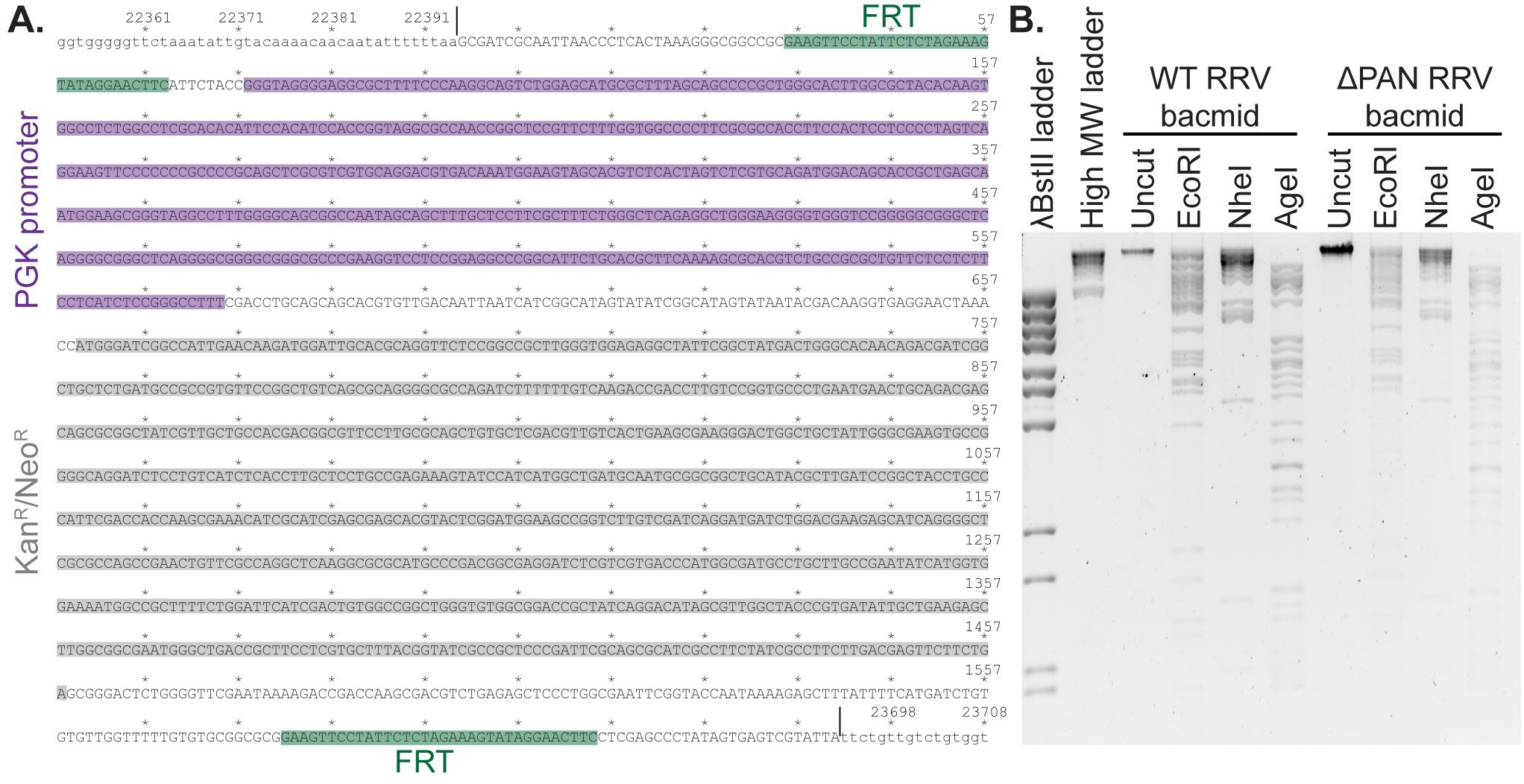

Supplement: S2 Fig — (A) Sequence of the DNA cassette inserted at the RRV PAN RNA locus. The entire PAN RNA sequence was deleted, including 140 bps upstream and 22 bp downstream that are necessary to express RRV PAN RNA [8]. A 1641-bp cassette was inserted between nucleotides 22394 and 23693 of the RRV genome reference sequence (accession number AF210726). Lower case: wild-type RRV bacmid sequence. Upper case: inserted DNA sequence including the PGK promoter (purple), kanamycin/neomycin resistance open reading frame (grey) and two FRT sites (green). (B) Ethidium bromide stained agarose gel of the RRV bacmid digested with indicated restriction enzymes. These analyses and sequencing revealed no apparent rearrangements between the wild-type (WT) and ΔPAN RRV bacmids. (TIF) [file ppat.1007389.s006.tif]

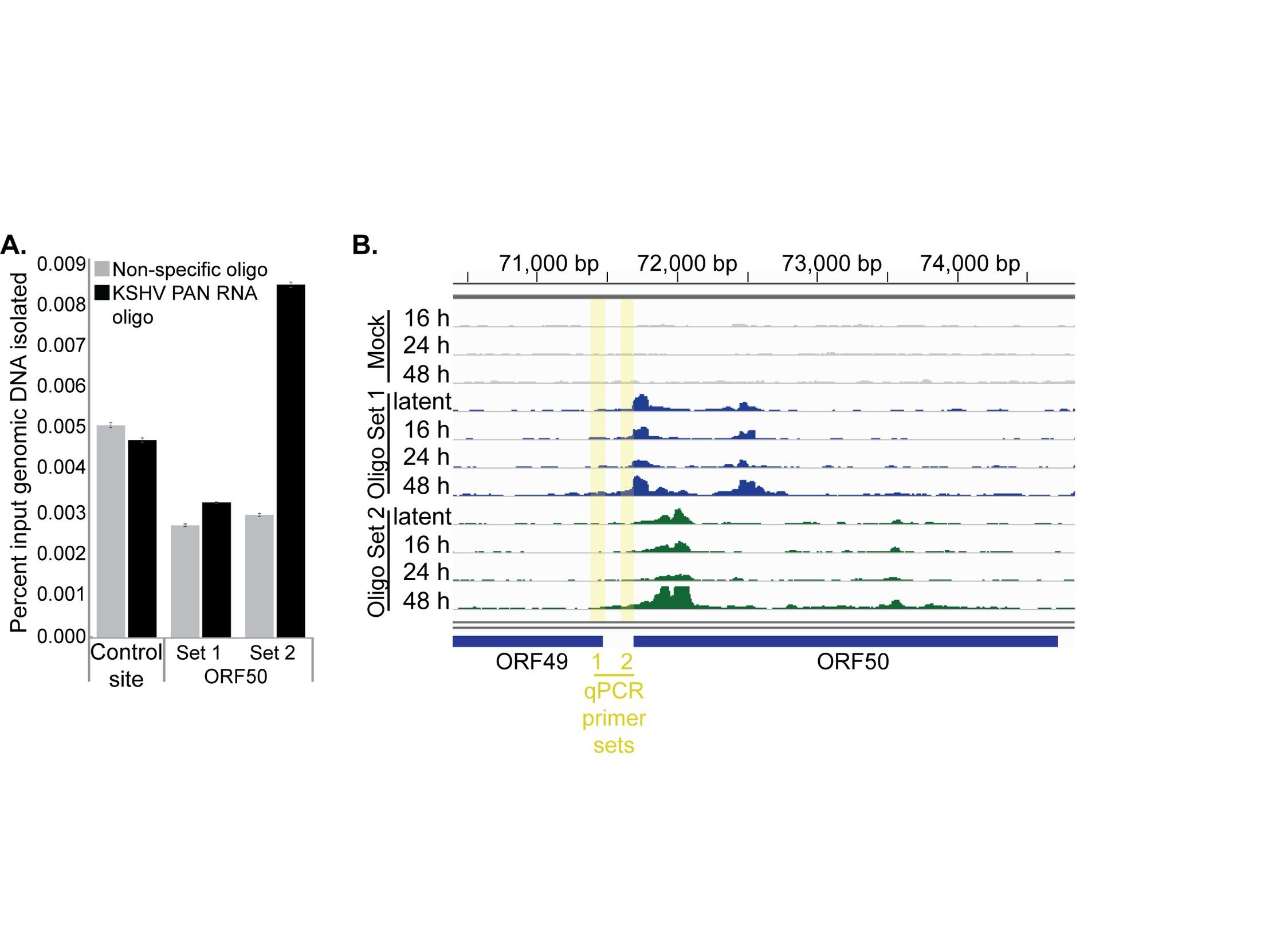

Supplement: S3 Fig — (A) qPCR of DNA isolated by KSHV PAN RNA CHART oligonucleotide set 1 (see Fig 3A and 3D) using published primers for the KSHV ORF50 promoter region [10]. (B) Genome browser view of the KSHV genome displaying KSHV CHART data from the region of the ORF50 promoter. qPCR primers overlapping the previously reported ChIRP enriched region [10] are shown in yellow. Set 1 (blue) and Set 2 (green) represent the KSHV PAN RNA CHART capture oligonucleotide sets. Mock denotes the sequencing data from a control CHART enrichment lacking a capture oligonucleotide. Sites of enriched DNA from the two KSHV CHART oligonucleotide sets do not overlap. (TIF) [file ppat.1007389.s007.tif]

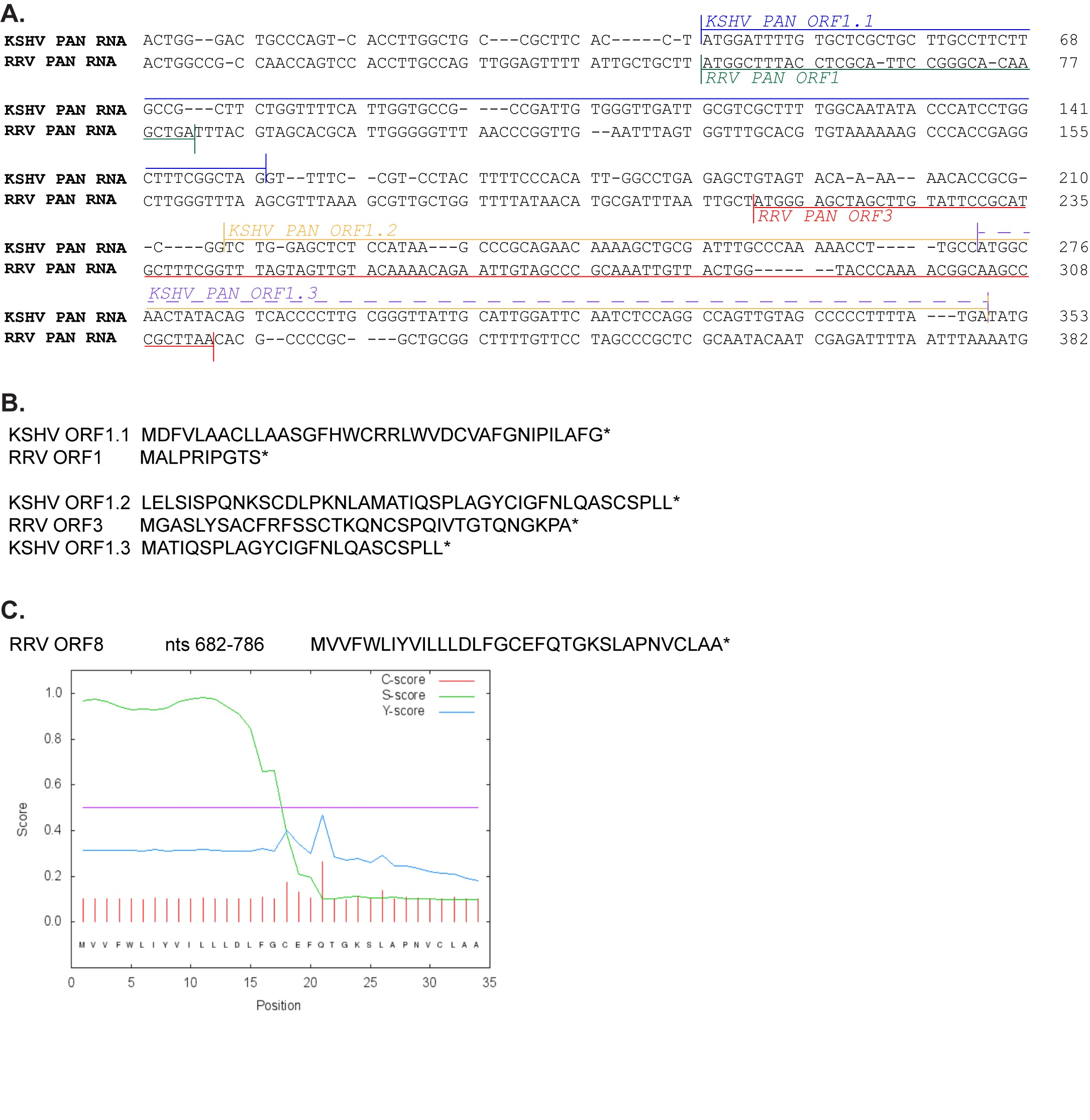

Supplement: S4 Fig — (A) Sequence alignment of a portion of the KSHV and RRV PAN loci. Candidate KSHV open reading frames identified by ribosome footprint profiling [48] are indicated (ORF1.1, ORF1.2 and ORF1.3). Two putative overlapping RRV open reading frames are indicated (ORF1 and ORF3). (B) Translation of indicated open reading frames. The peptide sequences are not conserved between viruses. (C) Peptide sequence of an open reading frame identified at residues 682–786 of RRV PAN RNA. This sequence is predicted to contain a secretory signal peptide by the SignalP 4.1 prediction program (www.cbs.dtu.dk/services/SignalP/). C-score predicts cleavage site, S-score predicts signal peptide location, Y-score is a combined cleavage site score that accounts for the location of the signal peptide. (TIF) [file ppat.1007389.s008.tif]

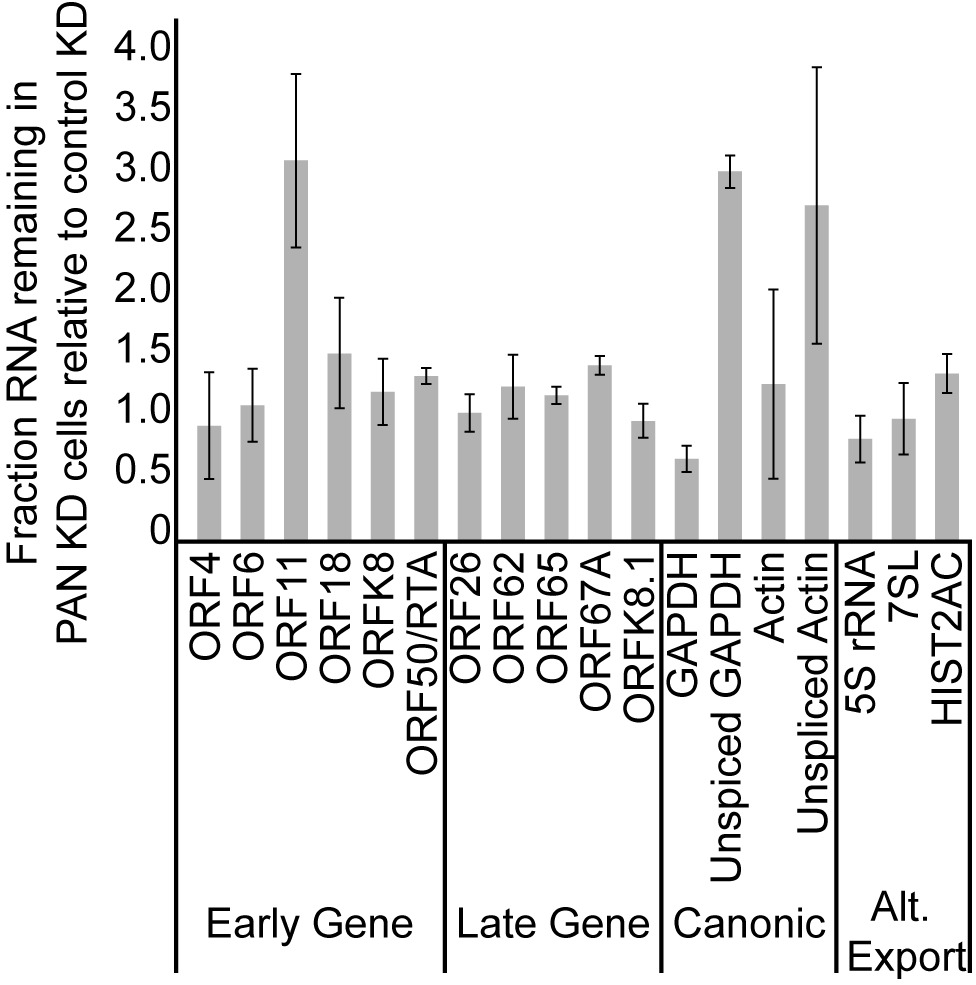

Supplement: S5 Fig — BCBL-1 TREx cells were electroporated with oligonucleotides antisense either to GFP mRNA (control KD) or to KSHV PAN RNA and then induced into the lytic phase with 1 μg/mL doxycycline. 48 h after induction, a subset of the cells was harvested for RT-qPCR quantification of transcript levels in PAN RNA knockdown cells, relative to those in control knockdown cells. The levels of KSHV ORF11 mRNA, and host unspliced GAPDH and unspliced actin transcripts increased upon PAN RNA knockdown. (TIF) [file ppat.1007389.s009.tif]

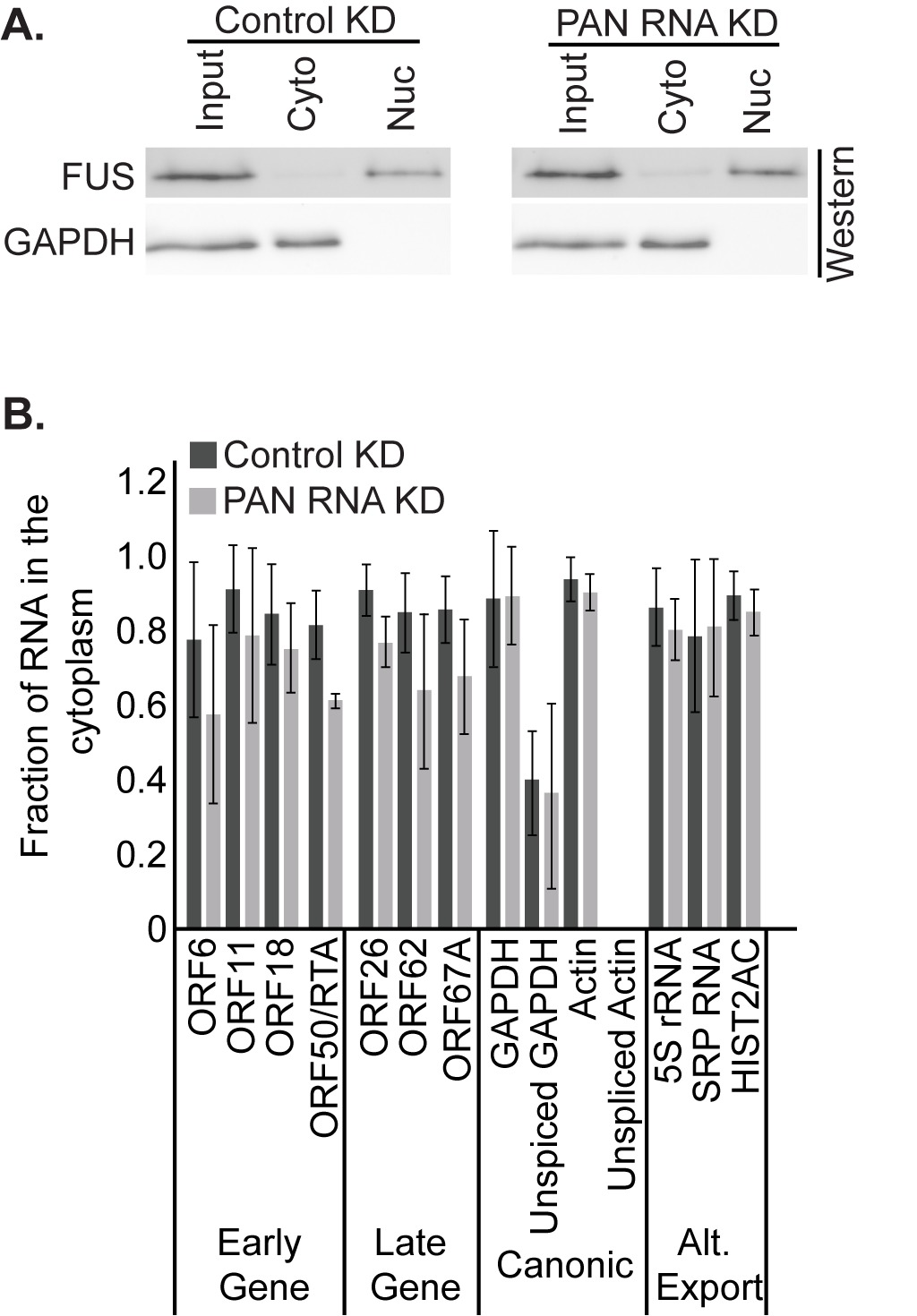

Supplement: S6 Fig — BJAB RRV cells were induced with 100 nM trichostatin-A (TSA) following electroporation with antisense oligonucleotides complementary to either GFP mRNA (control KD) or RRV PAN RNA (Oligo 493). After 40 h of lytic induction, cytoplasmic and nuclear fractions were isolated. (A) Western blot analysis assesses the purity of the cytoplasmic (GAPDH) and nuclear (FUS) fractions. (B) RT-qPCR analysis of fractionated BJAB-RRV cells. The fraction of each transcript present in the cytoplasm, relative to the nucleus is plotted. Despite extensive optimization of conditions, nuclei isolated from BJAB-RRV cells were prone to RNA leakage, which obscures the results of this analysis. Unspliced actin did not yield a signal above background samples lacking reverse transcription. (TIF) [file ppat.1007389.s010.tif]
